# No Chance to Survive: *Mo*-CBP_3_-PepII Synthetic Peptide Acts on *Cryptococcus neoformans* by Multiple Mechanisms of Action

**DOI:** 10.3390/antibiotics12020378

**Published:** 2023-02-12

**Authors:** Tawanny K. B. Aguiar, Felipe P. Mesquita, Nilton A. S. Neto, Francisco Í. R. Gomes, Cleverson D. T. Freitas, Rômulo F. Carneiro, Celso S. Nagano, Luciana M. R. Alencar, Ralph Santos-Oliveira, Jose T. A. Oliveira, Pedro F. N. Souza

**Affiliations:** 1 Department of Biochemistry and Molecular Biology, Federal University of Ceará, Fortaleza 60451-970, CE, Brazil; 2 Drug Research and Development Center, Department of Physiology and Pharmacology, Federal University of Ceará, Fortaleza 60430-275, CE, Brazil; 3 Department of Fisheries Engineering, Federal University of Ceará (UFC), Fortaleza 60451-970, CE, Brazil; 4 Laboratory of Biophysics and Nanosystems, Physics Department, Federal University of Maranhão, São Luís 65080-805, MA, Brazil; 5 Brazilian Nuclear Energy Commission, Nuclear Engineering Institute, Rio de Janeiro 21941-906, RJ, Brazil; 6 Laboratory of Nanoradiopharmacy, Rio de Janeiro State University, Rio de Janeiro 23070-200, RJ, Brazil

**Keywords:** alternative drugs, cryptococcosis, oxidative stress, synthetic peptides

## Abstract

Multidrug-resistant *Cryptococcus neoformans* is an encapsulated yeast causing a high mortality rate in immunocompromised patients. Recently, the synthetic peptide *Mo*-CBP_3_-PepII emerged as a potent anticryptococcal molecule with an MIC_50_ at low concentration. Here, the mechanisms of action of *Mo*-CBP_3_-PepII were deeply analyzed to provide new information about how it led *C. neoformans* cells to death. Light and fluorescence microscopies, analysis of enzymatic activities, and proteomic analysis were employed to understand the effect of *Mo*-CBP_3_-PepII on *C. neoformans* cells. Light and fluorescence microscopies revealed *Mo*-CBP_3_-PepII induced the accumulation of anion superoxide and hydrogen peroxide in *C. neoformans* cells, in addition to a reduction in the activity of superoxide dismutase (SOD), ascorbate peroxidase (APX), and catalase (CAT) in the cells treated with *Mo*-CBP_3_-PepII. In the presence of ascorbic acid (AsA), no reactive oxygen species (ROS) were detected, and *Mo*-CBP_3_-PepII lost the inhibitory activity against *C. neoformans*. However, *Mo*-CBP_3_-PepII inhibited the activity of lactate dehydrogenase (LDH) ergosterol biosynthesis and induced the decoupling of cytochrome *c* (Cyt c) from the mitochondrial membrane. Proteomic analysis revealed a reduction in the abundance of proteins related to energetic metabolism, DNA and RNA metabolism, pathogenicity, protein metabolism, cytoskeleton, and cell wall organization and division. Our findings indicated that *Mo*-CBP_3_-PepII might have multiple mechanisms of action against *C. neoformans* cells, mitigating the development of resistance and thus being a potent molecule to be employed in the production of new drugs against *C. neoformans* infections.

## 1. Introduction

Two decades have passed since the last new antifungal drug was released; however, the incidence of invasive infections by multidrug-resistant fungi has increased [1]. Three main antifungal classes are available to treat fungal infections: azoles, polyenes, and echinocandins. Nonetheless, their use includes limitations, such as the spectrum of activity, resistance, toxicity, drug-drug interactions, and poor bioavailability [2].

In a list of priority regarding fungal pathogens recently published by the World Health Organization (WHO) [3], *Cryptococcus neoformans* appears in the top three due to some reasons: (1) low susceptibility to current antifungal agents; (2) poorly understood resistance to drugs; and (3) high mortality rate ranging from 41% to 61% [3]. *C. neoformans* causes cryptococcosis, which mainly occurs in the lungs and leads to pneumonia but also affects the brain, causing meningitis, mainly in patients with a compromised immune system [3,4]. HIV^+^ patients, individuals with cancer, or taking any drug that weaken the immune system are predisposed to acquire cerebral cryptococcosis, which forms lesions known as cryptococcomas [3]. *C. neoformans* is naturally resistant to azole antifungals and the latest antifungal drug, Caspofungin, causing global health concerns [5,6]. Therefore, the search for new alternatives to treat cryptococcosis becomes emerging. In this context, antimicrobial peptides (AMPs) appear as a promise due to their mechanisms of action on the membrane [7].

Natural AMPs have limitations in their use and are generally cytotoxic. Moreover, the rational design of synthetic antimicrobial peptides (SAMPs) is an alternative to this problem. This approach is cost-effective for active short peptides [7,8,9]. Recently, our research group assessed the anticryptococcal potential of *Mo*-CBP_3_-PepII [10], a synthetic peptide bioinspired in the sequence of *Mo*-CBP_3_, a protein purified from *Moringa oleifera* seeds [11]. This study demonstrated that *Mo*-CBP_3_-PepII has an MIC_50_ of 25 µg mL^−1^ and induced membrane pore formation, DNA degradation, and apoptosis in cryptococcal cells. Additionally, it was reported that *Mo*-CBP_3_-PepII interacts with ergosterol in the membrane of *C. neoformans* favoring pore formation [10].

## 2. Results and Discussion

### 2.1. Mo-CBP_3_-PepII Induced ROS Overaccumulation in C. neoformans Cells

**Figure 1:**
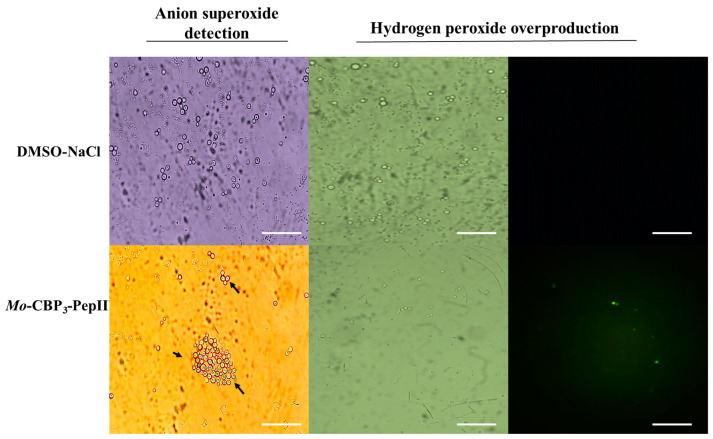
Detection of anion superoxide and hydrogen peroxide in *C. neoformans* cells. Light and fluorescence microscopies analysis to detect, respectively, the conversion of NBT (nitro blue tetrazolium chloride) into formazan (blue or cyan dots—black arrows) and the accumulation of hydrogen peroxide in *C. neoformans* cells treated with *Mo*-CBP_3_-PepII at MIC_50_ concentration. White bars indicate 100 µm.

All experiments in this study were carried out using the MIC_50_ concentration of 25 µg mL^−1^ of *Mo*-CBP_3_-PepII against *C. neoformans* as defined in our previous published study by Aguiar et al. [10], and all results presented statistical significance (*p* > 0.05) when compared treated and control. The *Mo*-CBP_3_-PepII-treated *C. neoformans* cells showed an accumulation of ROS, such as anion superoxide (O2^•−^) and hydrogen peroxide (H_2_O_2_) (Figure 1). The light microscopy analysis revealed the accumulation of O2^•–^ in *Mo*-CBP_3_-PepII-treated *C. neoformans* cells (Figure 1—blue or cyan dots—black arrows). As expected, the same was not observed in control cells (DMSO-NaCl) (Figure 1). The detection of H_2_O_2_ was carried out by fluorescence microscopy using 2′,7′ dichlorofluorescein diacetate (DCFH-DA, Sigma, St. Louis, MI, USA) assay. The green fluorescence on cells indicates the accumulation of H_2_O_2_ after treatment with *Mo*-CBP_3_-PepII. In contrast, DMSO-NaCl did not show the same (Figure 1—H_2_O_2_ panel).

ROS are inherent molecules of life as a byproduct of aerobic cell metabolism, and it is impossible to live without them. Moreover, ROS plays several essential roles in cell life, growth, development, defense, and signaling [12]. For pathogens such as fungi, ROS play critical roles in biofilm biogenesis, infection, virulence, and developmental process [13]. Even though these are sound effects of ROS, the induction of ROS overproduction is a mechanism of action employed by antimicrobial peptides to kill human pathogenic fungi [7,14,15,16,17]. What is essential to discuss is that ROS are good at low and controlled levels; if an external insult disturbs the ROS homeostasis, they could be lethal for cells. That is what antimicrobial peptides do.

Here, *Mo*-CBP_3_-PepII induced an uncontrolled accumulation of •O_2_^−^ and H_2_O_2_ (Figure 1). At higher levels, •O_2_^−^ and H_2_O_2_ could cause severe damage to cells by interacting and degrading critical cellular components such as DNA, proteins, and lipids, inducing membrane pore formation, apoptosis, and death [16,17,18,19,20]. For example, higher levels of •O_2_^−^ as demonstrated in Figure 1 led to DNA oxidation mediated by iron [20]. High levels of •O_2_^−^ increase free iron levels by releasing it from heme-oxidized proteins and enzyme clusters. The free iron interacts with DNA molecules oxidizing them and inducing fragmentation [20]. This result agrees with Aguiar et al. [10], that reported *Mo*-CBP_3_-PepII induced DNA fragmentation in *C. neoformans* cells.

In the case of H_2_O_2_, higher levels of H_2_O_2_ drive the lipid peroxidation in the membrane and, thus, pore formation [18]. Recently, it was reported that two synthetic peptides, *Rc*Alb-PepII and PepGAT, lost the ability to induce pore formation in the *C. neoformans* membrane in the presence of an antioxidant agent where there was no H_2_O_2_ accumulation. This result suggests both peptides induce pore formation by ROS-dependent pathway [21]. Besides damaging the membrane, higher levels of H_2_O_2_ could also result in a higher level of oxidized protein and loss of activity. Proteomic analysis of *Klebsiella pneumoniae* cells in the presence of a synthetic peptide *Mo*-CBP_3_-PepI, which induced higher levels of H_2_O_2_, revealed an increase in the accumulation of proteins involved in the repair of oxidized proteins [22]. These data suggest that *K. pneumoniae* cells are trying to fix the damaged proteins by H_2_O_2_. Recently, Chen et al. [23] reported by RNA-seq that many genes involved in antioxidative stress have reduced expression in *C. neoformans* cells after the exposition to the synthetic peptide Sparamosin_26–54_ compared to control cells leading to the accumulation of ROS.

### 2.2. Mo-CBP_3_-PepII Affects the Activity of Redox System Enzymes

**Figure 2:**
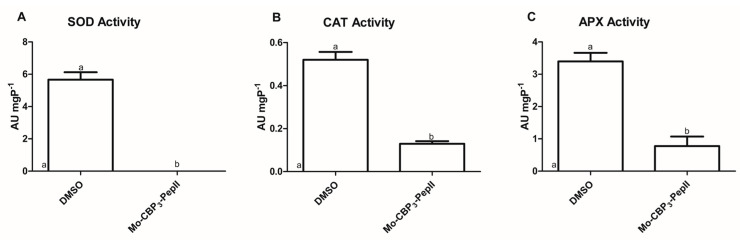
The activity of enzymes involved in ROS in *C. neoformans* cells after treatment with *Mo*-CBP_3_-PepII. (**A**) SOD activity that converts •O_2_^−^ into H_2_O_2_, (**B**) CAT activity that converts H_2_O_2_ into H_2_O in O_2_, and (**C**) APX activity that converts H_2_O_2_ into H_2_O in O_2_ using ascorbate as electron donor. The activity of enzymes was tested in *C. neoformans* cells treated (*Mo*-CBP_3_-PepII) and non-treated (DMSO) with the synthetic peptide. The different lowercase letters indicate statistical significance at *p* > 0.05.

Aguiar et al. [10] revealed *Mo*-CBP_3_-PepII forms a 6-kDa pore in the membrane of *C. neoformans*. So, it was reasoned that *Mo*-CBP_3_-pepII could move by this pore and interfere with the activity of some cytoplasmic enzymes involved in ROS metabolism, leading to ROS accumulation, as shown in Figure 1. As discussed above, ROS are essential to life but only if controlled to safer levels [12]. The safer levels of ROS were maintained by two mechanisms: enzymatic and non-enzymatic [24]. The enzymatic is mediated by scavenger enzymes that either convert highly unstable ROS molecules to more stable ones or completely oxidize them to H_2_O and O_2_ [24]. Here, the activity of three enzymes, SOD, CAT, and APX, was analyzed (Figure 2).

The first enzyme analyzed was SOD because it is involved in the conversion of •O_2_^−^ to H_2_O_2_ [25]. In control *C. neoformans* cells, the SOD activity was 5.67 AU mgP^−1^. In contrast, no activity was detected in *Mo-*CBP_3_-PepII-treated cells (Figure 2A). In addition, two other enzymes were analyzed, CAT and APX, both involved in converting H_2_O_2_ into H_2_O in O_2_ [26,27]. Regarding the CAT activity, control cells of *C. neoformans* presented an activity of 0.52 AU mgP^−1^, and *Mo-*CBP_3_-PepII-treated cells showed a value of 0.121 AU mgP^−1^ (Figure 2B), representing a decrease of four times in CAT activity after treatment with *Mo*-CBP_3_-PepII. The APX activity from *C. neoformans* cells was also affected by *Mo-*CBP_3_-PepII (Figure 2C). *Mo-*CBP_3_-PepII (0.77 AU mgP^−1^) induced a reduction of 4.5-fold in APX activity compared to control cells (3.40 AU mgP^−1^) (Figure 2C).

As demonstrated in Figure 2, *Mo*-CBP_3_-PepII induced alteration in the activity of enzymes involved in the ROS scavenger process. By an unclear mechanism, *Mo*-CBP_3_-PepII insults the balance of ROS in *C. neoformans* cells. First, the inhibition of SOD activity (Figure 2A) agrees with the accumulation of •O_2_^−^ (Figure 1). Second, the reduction in the activity of CAT and APX let high the levels of H_2_O_2_ (Figure 2B,C). The number of studies showing such alteration in redox enzymes is scarce. Two synthetic peptides, *Rc*Alb-PepII and *Rc*Alb-PepII, could also completely inhibit the SOD activity and reduce CAT and APX activities in *C. neoformans* cells, leading to ROS accumulation [21]. In another study, not with peptides but with an antifungal protein, *Mo*-CBP_2_, purified from *Moringa oleifera* seeds, presented perturbation in the activity of those enzymes in *Candida albicans* [28].

This scenario strongly corroborates the previous results of *Mo*-CBP_3_-PepII [10]. Recently, Aguiar et al. [10] *Mo*-CBP_3_-PepII induced DNA fragmentation and apoptosis in *C. neoformans* cells. Altogether, previous results and those presented here showed a high accumulation of ROS in *C. neoformans* cells that could drive DNA fragmentation and apoptosis.

### 2.3. Ascorbic Acid (AsA) Affects the Anticryptococcal Potential of Mo-CBP_3_-PepII

**Figure 3:**
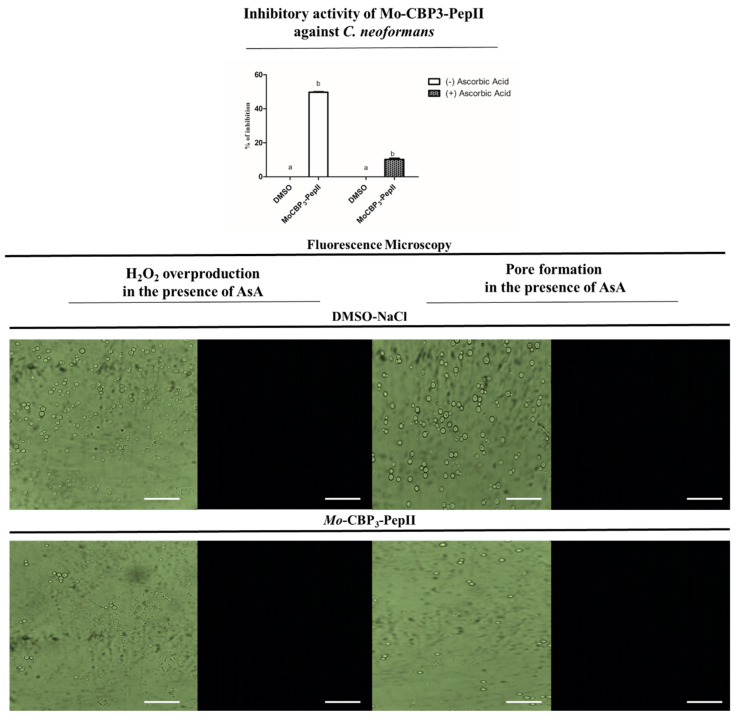
Effect of the antioxidant agent, ascorbic acid, in the activity of Mo-CBP_3_-PepII against *C. neoformans*. Graphic: Inhibitory activity of Mo-CBP_3_-PepII at MIC_50_ concentration (25 µg mL^−1^) against *C. neoformans* in the presence and absence of 10 mM of ascorbic acid. Fluorescence Microscopy analysis showed no detection of H_2_O_2_ and no pore formation in the cells of *C. neoformans* in the presence of 10 mM of ascorbic acid. Bars indicate 100 μm. The different lowercase letters in (graphic) indicate statistical significance at *p* > 0.05.

Figure 1 and Figure 2 revealed *Mo*-CBP_3_-PepII induced the overaccumulation of ROS by negatively modulating the activity of scavenger enzymes. These results correspond to previous damage caused by *Mo*-CBP_3_-PepII in *C. neoformans* cells [10]. Based on that, one question emerged: is the activity of *Mo*-CBP_3_-PepII ROS-dependent? To answer this question, *Mo*-CBP_3_-PepII was assayed against *C. neoformans* in the presence of 10 mM of AsA. At the concentration of 25 µg mL^−1^, *Mo*-CBP*3*-PepII reaches the MIC_50_ without AsA (Figure 3—graphic). In the presence of 10 mM of AsA, the anticryptococcal activity of *Mo*-CBP_3_-PepII presented a significant reduction of 40% (*p* > 0.05) (Figure 3—graphic).

Aguiar et al. [10] reported that *Mo*-CBP_3_-PepII could induce pore formation in membranes of *C. neoformans* cells, as revealed by the propidium iodide (PI) uptake assay. In the presence of AsA, fluorescence microscopy revealed no H_2_O_2_ accumulation and pore formation (Figure 3), indicating that *Mo*-CBP_3_-PepII lost the ability to induce pore formation. This result suggests the ability of *Mo*-CBP_3_-PepII to induce pore formation is H_2_O_2_-dependent.

As happened to ROS, pore formation is also a common mechanism of action played by peptides because it could result from ROS accumulation and lipid peroxidation or the direct action of peptides in the membrane [18,29,30]. These data suggest the activity and mechanism of action of *Mo*-CBP3-PepII are dependent on ROS accumulation. This result is different from others presented by peptides RcAlb-PepIII and PepKAA [21], where the ability of pore formation is independent of ROS.

It seems that *Mo*-CBP_3_-PepII first interacts with *C. neoformans* cells interfering with the ROS scavenger enzyme activity leading to the accumulation of ROS and, thus, several other damages to *C. neoformans,* driving them to death.

### 2.4. Mo-CBP_3_-PepII Interferes in the Biosynthesis of Ergosterol

**Figure 4:**
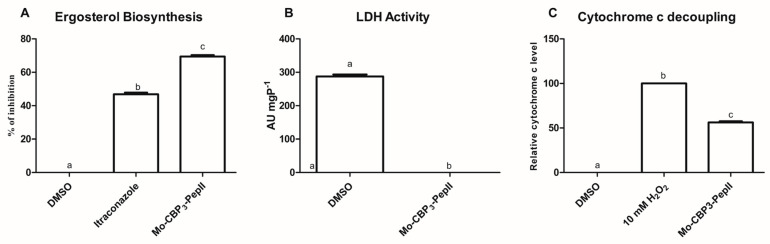
Mo-CBP_3_-PepII interferes with vital cellular processes for *C. neoformans* cells. (**A**) inhibition of the biosynthesis of ergosterol, (**B**) lactate dehydrogenase activity, and (**C**) release of Cyt c from the mitochondrial membrane. The different lowercase letters in (graphic) indicate statistical significance at *p* > 0.05.

Ergosterol is a fungal membrane component, and its primary function is acting as a stabilizer [31]. The biosynthesis of ergosterol occurs in the cytoplasm of the cell and is transported to the membrane [31]. Aguiar et al. [10] demonstrated that *Mo*-CBP_3_-PepII was able to bind to ergosterol. Therefore, we experimented with evaluating the ability of *Mo*-CBP_3_-PepII to inhibit ergosterol synthesis (Figure 4A). As expected, in control with DMSO-NaCl, there was no inhibition of ergosterol synthesis. The positive control for inhibition, itraconazole (ITR), inhibited 47.65% of ergosterol biosynthesis (Figure 4A). Meanwhile, *Mo*-CBP_3_-PepII inhibited the ergosterol biosynthesis in 72% (Figure 4A), a value 1.5 times higher than ITR.

By inhibiting the biosynthesis of ergosterol, *Mo*-CBP_3_-PepII destabilizes the membrane facilitating the pore formation on the membrane leading to loss of cytoplasmic content and cell death. *Mo*-CBP_3_-PepII targets ergosterol in two ways: interacting with it in the membrane [10] or inhibiting its synthesis in *C. neoformans* cells (Figure 4A). In agreement with our data, Chen et al. [23] reported a reduction in the gene expression involved in ergosterol biosynthesis in *C. neoformans* cells after treatment with Sparamosin_26–54_.

### 2.5. Energetic Metabolism Is Affected in C. neoformans Treated with Mo-CBP_3_-PepII

Still looking for possible cytoplasmic targets of *Mo*-CBP_3_-PepII, the lactate dehydrogenase (LDH) activity was assayed in *C. neoformans* cells after contact. The activity of LDH was wholly inhibited in cells treated with *Mo*-CBP_3_-PepII (MIC_50_) (Figure 4B). As expected, control cells did not suffer any alteration in LDH activity (Figure 4B).

LDH is an essential enzyme in carbohydrate metabolism because it is involved in the regeneration of NAD^+^ from NADH produced in the glycolysis pathway. This reaction maintains a high ratio of NAD^+^/NADH in the cytoplasm, favoring glycolysis [32]. By inhibiting the LDH activity, *Mo*-CBP_3_-PepII interferes in the regeneration of NAD^+^, which is essential for glycolysis to still work. The inhibition of LDH can jeopardize all energetic metabolism in *C. neoformans* cells.

Another parameter of energetic metabolism evaluated was the decoupling of Cyt c from the mitochondrial membrane (Figure 4C). In cells treated with *Mo*-CBP_3_-PepII (MIC_50_), there was a significant increase of 51% of Cyt *c* content in the cytoplasm. The positive control H_2_O_2_ promotes 100% Cyt *c* release from mitochondrial to cytoplasm (Figure 4C), and DMSO did not promote any release of Cyt c. At that time, *Mo*-CBP_3_-PepII is interfering with energy production in mitochondria. The release of Cyt c from the mitochondrial membrane impairs the complete function of the electron transport chain (ETC) in the mitochondrion leading to a depletion in ATP levels [33]. These analyses suggest *Mo*-CBP_3_-PepII has targets within *C. neoformans* cells and might reach the cytoplasm and other organelles.

One fact is *Mo*-CBP3-PepII affects the energy production of *C. neoformans* cells in both stages in the cytoplasm and mitochondria. Energy is critical for any organism to cope with stresses of any kind, for example, to produce defense proteins [34]. Here, we showed that *Mo*-CBP3-PepII imposes different types of stress on *C. neoformans* cells and that shutting down the energy production mitigates the chances of *C. neoformans* fighting back.

The role in ETC is the canonical function of Cyt c. However, its role goes beyond that [33]. Cyt c is also crucial for the health of mitochondria. The decoupling of Cyt c from mitochondrial membranes signals mitochondrion misfunction and thus starts cell apoptosis [33]. Therefore, by inducing the release of o Cyt c from the mitochondrial membrane, *Mo*-CBP_3_-PepII induces *C. neoformans* cells to enter apoptosis. Aguiar et al. [10] revealed that *Mo*-CBP_3_-PepII induced apoptosis in *C. neoformans* cells corroborating the results reported here. Another study noticed a reduction in the genes involved in oxidative phosphorylation in *C. neoformans* after treatment with the synthetic peptide Sparamosin_26–54_ [23]. In that study, authors revealed a reduction in the expression of 25 genes involved in energy production by oxidative phosphorylation suggesting the failure of *C. neoformans* cells to produce energy to cope with the insult imposed by Sparamosin_26–54_ [23]

### 2.6. Mo-CBP_3_-PepII Promotes Several Changes in the Cell Structure of C. neoformans

**Figure 5:**
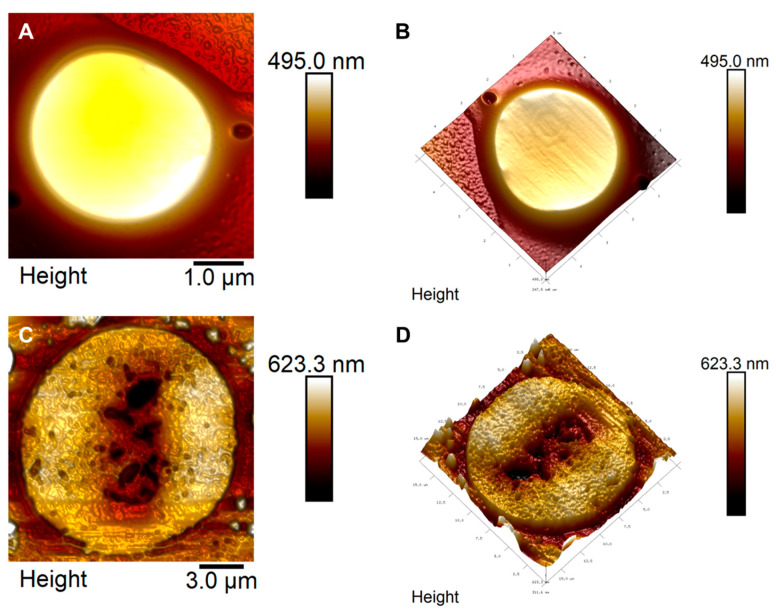
Atomic force microscopy (AFM) images showing damage in *C. neoformans* cells induced by *Mo*-CBP_3_-PepII. (**A**,**B**) Control cell of *C. neoformans* treated with DMSO. (**C**,**D**) The cell of *C. neoformans* treated with Mo-CBP3-PepII.

Atomic force microscopy (AFM) analysis revealed the effects that *Mo*-CBP_3_-PepII induces in the structure of *C. neoformans* cells. The control showed a cryptococcal cell in its morphology with no damage (Figure 5A,B), healthy and continuous cytoplasmatic membrane, homogenous cytoplasm, and no damage to the capsule. By contrast, *C. neoformans* treated with *Mo*-CBP_3_-PepII showed several damages in its structure, such as damage to the membrane, cell wall thickness, damage to fungal capsules, wrinkled cytoplasm, and roles across the whole cells (Figure 5C,D—brown dots). Additionally, it is possible to see that *Mo*-CBP_3_-PepII-treated cells have a diameter and height and hence higher volume than control cells indicating treated cells are bigger than control cells (Figure 5C,D). This result suggests that *Mo*-CBP_3_-PepII induced pore formation in the *C. neoformans* membrane by changing its permeability. In that case, cells cannot control the osmotic potential. Additionally, 3D images (Figure 5D) showed that *C. neoformans* cells treated with *Mo*-CBP_3_-PepII are flattened, presenting structures such as depression in the middle of the cell.

Recently, the morphology of *C. neoformans* treated with synthetic peptides was revealed by scanning electron microscopy [10]. In this study, authors revealed several damages to the cell structure; in some cases, those are similar to data presented in this study, but AFM analysis brought more details on the effect of *Mo*-CBP_3_-PepII on *C. neoformans* cells. Similar AFM data on *C. neoformans* cells were reported by Ishida et al. [35] using silver nanoparticles. In the study, the authors reported that *C. neoformans* cells presented damage in the cell wall, cell capsule, and membrane.

### 2.7. Proteomic Profile of C. neoformans Treated with Mo-CBP_3_-PepII

#### 2.7.1. Overview

Proteomic analysis is an important technique to see the whole picture of a cell by evaluating the protein profile after treatment with peptides [36,37,38,39,40]. Proteomic analysis has already been employed to study the response of resistant pathogens such as *Clostridioides difficile* [38]. Regarding *C. neoformans*, proteomic analysis was employed to understand the changes in protein profile during the transition from planktonic to biofilm lifestyle [41]. As far as we know, no study has employed proteomics to analyze the *C. neoformans* response to antimicrobial peptides, reinforcing the pioneering of our work.

**Figure 6:**
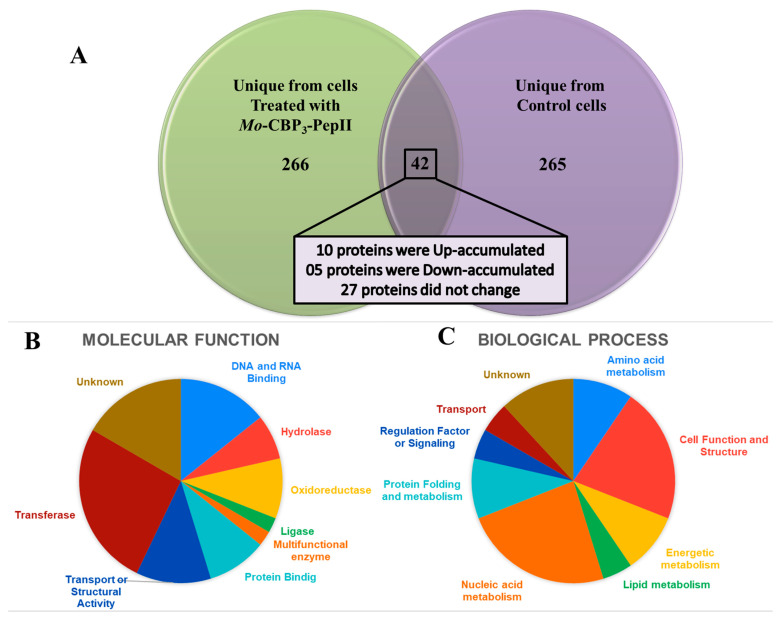
Differentially accumulated proteins in *C. neoformans* cells treated with Mo-CBP3-PepII. In (**A**), the Venn diagram highlights the differential distribution of proteins in treated and non-treated cells unique from each group and the overlapping protein found in both groups with differential accumulation. (**B**) Proteins are classified according to molecular function. (**C**) Proteins are classified according to biological processes.

As shown above, *Mo*-CBP_3_-PepII presented different mechanisms against *C. neoformans* cells. However, all of those are despite only the cell structure itself. After that, one new question arises: is *Mo*-CBP_3_-PepII able to change the protein profile of *C. neoformans*? A proteomic analysis was performed to obtain the answer (Figure 6, Table 1, and Supplementary Tables S1 and S2).

Five hundred seventy-three proteins were identified (Figure 6A, Table 1, and Supplementary Tables S1 and S2). Of these, 265 were exclusively identified in control cells (Supplementary Table S1); 266 were exclusively detected in *Mo*-CBP_3_-PepII-treated cells (Supplementary Table S2); and 42 were detected in both groups (Figure 6A and Table 1).

The proteins identified in both groups were called overlapping proteins. For these proteins, fold-change was calculated based on the intensity of peptides for proteins in *Mo*-CBP_3_-PepII-treated/control cells [42]. Proteins with a fold-change value ≥1.5 (*p* < 0.05, Tukey’s test) [42] were called up-accumulated (increase in abundance). Proteins with a fold-change value of ≤0.5 (*p* < 0.05, Tukey’s test) [42] were called down-accumulated (decrease in abundance). Proteins with a fold-change value ranging from 0.5 to 1.5 (*p* < 0.05, Tukey’s test) were considered they did not change in abundance [42] (Table 1). Among the overlapping proteins, 10 were up-accumulated, and five were down-accumulated in *Mo*-CBP_3_-PepII-treated cells compared to control cells (Figure 6A and Table 1). Twenty-seven overlapping proteins did not change their accumulation in response to *Mo*-CBP_3_-PepII treatment in *C. neoformans* cells (Figure 6A and Table 1).

The overlapping proteins were classified based on gene ontology for molecular function and biological process (Figure 6B,C). Regarding the molecular function, proteins were classified into nine groups: DNA and RNA Binding (14.28%), Hydrolase (7.14%), Oxidoreductase (9.52%), Ligase (2.38%), Multifunctional enzyme (2.38%), Protein Binding (9.52%), Transport or Structural Activity (11.90%), Transferase (26.19%), and Unknown (16.66%) (Figure 6B, Table 1).

The biological process also revealed nine groups of proteins: Amino acid metabolism (9.52%), Cell Function and Structure (21.42%), Energetic metabolism (9.52%), Lipid metabolism (4.76%), Nucleic acid metabolism (23.80%), Protein folding (9.52%), Regulation Factor or Signaling (4.76%), Transport (4.76%), and Unknown (11.90%) (Figure 6C, Table 1).

#### 2.7.2. DNA and RNA Binding Proteins

In this group, two isoforms, DNA topoisomerase I and DNA topoisomerase II, were exclusively detected in the *C. neoformans* cells treated with *Mo*-CBP_3_-PepII (Supplementary Table S2). DNA topoisomerases are proteins that solve topological problems in DNA molecules, such as supercoiling and catenation [43,44]. Topoisomerases are a well-known target for antifungal drugs such as echinocandins [43]. Despite their function in regulating DNA torsion, topoisomerases are also involved in the repair of damaged DNA. The exclusive detection of two isoforms of topoisomerases in treated cells indicates *Mo*-CBP_3_-PepII [45].

Additionally, Pommier et al. [45] reported topoisomerase I is induced during cellular stress to prevent DNA hence cell death mediated by apoptosis [45]. The increase in topoisomerase I suggests the *Mo*-CBP_3_-PepII is inducing damage to DNA. It is trustworthy to notice that Aguiar et al. [10] revealed that *Mo*-CBP_3_-PepII induced DNA fragmentation and apoptosis; maybe these processes are related to apoptosis in *C. neoformans* cells induced by *Mo*-CBP_3_-PepII.

Another protein only identified in *Mo*-CBP3-PepII-treated *C. neoformans* cells was the Mitochondrial escape protein 2 (MET2) (Supplementary Table S2). MET2 protein controls the escape of mitochondrial DNA during stress to prevent degradation [46,47]. Once mitochondria suffer any external insult that could lead to DNA damage, the MET2 proteins are involved in the protection, repair, or even DNA escape to prevent worse damage [46,47]. Here, the increase in the abundance of MET2 protein only in treated cells suggests that *Mo*-CBP_3_-PepII attacks mitochondria, which follows the results above-mentioned that *Mo*-CBP_3_-PepII induces the decoupling of Cyt c from the mitochondrial membrane (Figure 4C).

However, another protein that deserved attention, the clustered mitochondria protein (CMP), was only detected in the control cells and not in treated cells (Supplementary Tables S1 and S2). The CMP in fungi and many other eukaryotic organisms is essential for mitochondrial health and functioning [48,49,50]. The absence of CMP leads to misfunction of mitochondria interfering with the normal function of the cell. This result indicates that *Mo*-CBP_3_-PepII caused several damages to the mitochondrion, reflecting the whole *C. neoformans* cell.

#### 2.7.3. Ligase- and Amino Acid Metabolism-Related Proteins

In the ligase group, one protein was found in both treated and non-treated *C. neoformans* cells, the carbamoyl-phosphate synthase (Table 1). The fold-change value, 0.07, indicates that carbamoyl-phosphate synthase (CPS) decreased abundance after treatment with *Mo*-CBP_3_-PepII (Table 1). The CPS protein is involved in the biosynthesis of arginine [51,52]. Arginine is an essential amino acid involved in protein synthesis and many other physiological and biochemical processes [52]. Liu et al. [52] reported that a CPS-mutant of pathogenic fungi *Magnaporthe oryzae* cannot produce arginine and affect the pathogenicity and development processes. Here, reducing CPS protein in *C. neoformans* cells could lead to arginine depletion, affecting vital cellular processes and, ultimately, death. Another protein, the Arginine biosynthesis bifunctional protein, in which two isoforms were exclusively from control cells, reinforces the hypothesis that *Mo*-CBP_3_-PepII interferes in arginine biosynthesis in *C. neoformans* cells (Supplementary Tables S1 and S2).

Proteomic analysis revealed many proteins involved in amino acid metabolism were only detected in control cells (Supplementary Table S1), such as Alanine-tRNA ligase, histidine biosynthesis trifunctional protein, C-1-tetrahydrofolate synthase, glycine dehydrogenase. After treatment with the peptide, the decrease in abundance in all those proteins indicates a shutdown in protein synthesis [53]. The inability of the cell to produce proteins after the treatment with a drug causes severe forms of stress and inhibits cells from responding to stresses leading to death [53]. An antifungal drug called sordarin inhibits protein synthesis in pathogenic yeasts [54]. However, many yeasts, including *C. neoformans,* are intrinsically resistant to sordarin, making this drug useless for treatment. Currently, there are no drugs available that affect the protein synthesis of fungi, which makes more attractive the effect of *Mo*-CBP_3_-PepII in inhibiting the protein synthesis of *C. neoformans*.

#### 2.7.4. Oxidoreductase-Related Proteins

In this group, one protein, D-2-hydroxyglutarate-pyruvate transhydrogenase, presented a fold-change value indicating its up-accumulation of it after exposure of *C. neoformans* cells to *Mo*-CBP_3_-PepII (Table 1). The D-2-hydroxyglutarate-pyruvate transhydrogenase is mainly involved in the metabolism of lactate and oxidation of NADH to produce NAD^+^ in the absence of LDH activity [55,56]. In *Saccharomyces cerevisiae,* the D-2-hydroxyglutarate-pyruvate transhydrogenase, also known as minor D-LDH, catalyzes the conversion of D-2-hydroxyglutarate into α-ketoglutarate using FAD^+^ as a cofactor and pyruvate as the donor of electrons producing lactate. The α-ketoglutarate in the cytosol is rapidly converted into 2-hydroxyglutarate, which is degraded in the cytosol by D-2-hydroxyglutarate-pyruvate transhydrogenase consuming NADH restoring NAD^+^ [55,56].

As shown before, the activity of LDH is inhibited in cells after treatment with *Mo*-CBP_3_-PepII interfering in NAD^+^ restoring and impairing the glycolysis to keep stand. The up-accumulation of D-2-hydroxyglutarate-pyruvate transhydrogenase suggests the *C. neoformans* cells are trying to find a way to continue with the glycolysis pathway by employing an alternative way to maintain acceptable levels of NAD^+^ essential to the glycolysis pathway.

#### 2.7.5. Protein Binding-Related Proteins

By analyzing proteins in this group, an unexpected result was seen. Many protein components of the 26S proteasome, such as 26S proteasome regulatory subunit RPN1, 26S proteasome regulatory subunit RPN5, U3 small nucleolar RNA-associated, ERAD-associated E3 ubiquitin-protein, Uba3-binding protein but2, and Ubiquinone biosynthesis protein, were only detected in control cells, being absent in treated cells (Supplementary Tables S1 and S2). In eukaryotic cells, the proteasome is a multicomplex enzymatic system that plays a role in protein turnover and many other cell processes, such as development, growth, division, cell-cycle progression, and defense [57,58,59]. The misfunction of proteasome could trap cells in a cell-cycle arrest and, consequently, apoptosis [58].

Here, the proteomic analysis revealed many proteasome subunits present in control cells, which is essential to its function, disappeared after the treatment with *Mo*-CBP_3_-PepII (Supplementary Table S2). This result suggests that *C. neoformans* do not have a functional proteasome and could start apoptosis. This idea agrees with the results of Aguiar et al. [10], where *Mo*-CBP_3_-PepII induces apoptosis in *C. neoformans* cells.

Another protein unique from the control cell was ASI1 (Supplementary Table S1). The ASI1 is a nuclear inner membrane-attached protein that regulates gene expression [60,61,62]. The protein ASI1 is involved in the functional folding of a group of transcription factors known as Stp proteins. In the absence of ASI1, unprocessed forms of Stp proteins were produced, leading to cell failure in control gene expression, involved in ribosomal RNA (rRNA) production [62]. So, somehow *Mo*-CBP_3_-PepII induces alteration in the expression of rRNA and consequently reduces ribosomes in *C. neoformans* treated cells. This result agrees with the shutting down in protein synthesis discussed above.

#### 2.7.6. Transferase-Related Proteins

Eleven proteins in this group presented overlapping in the treated and control cells (Table 1). Of these, six proteins were up-accumulated, and five did not change. First is the atypical kinase COQ8, with a fold-chance value of 1.81 (Table 1). The atypical kinase COQ8 is a mitochondrial protein involved in coenzyme Q (CoQ) biosynthesis [63,64]. CoQ is a molecule involved in at least two critical processes in eukaryotic cells: (1) acting as electron transport in ETC and (2) working as an antioxidant [63,64]. Here, we reasoned the increase in atypical kinase COQ8 to increase the CoQ levels is a response of *C. neoformans* cells to two stresses imposed by *Mo*-CBP_3_-PepII. First, high production of CoQ could be involved in a repair process of ETC, which is affected by the decoupling of Cyt c from the mitochondrial membrane (Figure 4C). As discussed above, the release of Cyt c from mitochondria jeopardizes the ETC and depletes the ATP synthesis. Second, higher CoQ levels might be associated with its antioxidant activity as a defense against ROS overaccumulation induced by *Mo*-CBP_3_-PepII (Figure 1).

The threonylcarbamoyl-AMP synthase presented a fold-change of 8.81 (Table 1), one of the higher evaluated in *C. neoformans* cells treated with *Mo*-CBP_3_-PepII. The threonylcarbamoyl-AMP synthase is an important enzyme involved in the production of threonylcarbamoyl-AMP, a central metabolite essential for biosynthesis L-threonine and a universal tRNA nucleoside N6-threonylcarbamoyl adenosine involved in the maturation of tRNA [65,66]. The increase in threonylcarbamoyl-AMP synthase might be an attempt of *C. neoformans* cells to overcome the reduction in protein synthesis, as suggested above.

The enzyme (2E.6E)-farnesyl diphosphate synthase (FPPS) presented a fold-change of 11.12 in treated cells compared to control cells (Table 1). The FPPS protein is a crucial enzyme involved in a central biochemical pathway for eukaryotic cells, the isoprenoid biosynthesis pathway [67,68,69,70]. The isoprenoid biosynthesis produces sesquiterpenes that supply the production of many essential metabolites such as ubiquinone, dolichols, and sterols; in our case, for fungi, the participation in sterols biosynthesis leads to ergosterol biosynthesis [67,68,69,70]. Here, it was reported that *Mo*-CBP_3_-PepII inhibits 60% of the biosynthesis of ergosterol, which in turn compromises the health of cellular membranes [68,69,70]. Altogether, this high fold-change value of FPPS led us to hypothesize that *C. neoformans* is trying to compensate for the inhibition of ergosterol biosynthesis to alleviate the damage to the membrane keeping it healthy and functional.

Another protein that was up-accumulated in the treated cells compared to the control was the spindle assembly checkpoint kinase (SAC). The SAC is a signal protein that indicates the mistaken attachment of the mitotic spindle to the kinetochores of chromosomes [71,72,73]. The SAC protein is vital during the checkpoint in the cell cycle. SAC protein is essential to check the correct position of chromosomes during the transition of metaphase-to-anaphase. If something goes wrong in this process, the SAC protein accumulates and negatively regulates the CDC20 (cell division cycle protein 20 homologs), inhibiting cell division from preventing duplicated chromosome separation. The cell cycle is stopped until chromosomes are correctly aligned to the spindle [71,72,73]. The higher accumulation of SAC proteins in *C. neoformans* cells treated with *Mo*-CBP_3_-PepII indicates an inhibition of the cellular cycle, decreasing the rate of cell division and, thus, in the case of *C. neoformans*, infection and pathogenicity [71,72,73].

#### 2.7.7. Transport or Structural Activity-Related Proteins

Most of the overlapping proteins in this group decrease in accumulation in treated/control cells (Table 1), including low-affinity methionine permease. Methionine is an essential proteinogenic amino acid [74,75]. As an essential amino acid, cells cannot synthesize methionine and must obtain it from the environment. To accomplish this, cells use transports attached to the membrane to collect amino acids [74,75]. The low-affinity methionine permease transporter is used by *C. neoformans* cells to collect methionine from the environment. The *Mo*-CBP_3_-PepII-treated *C. neoformans* cells presented a decrease in abundance of this transporter, leading to a decrease in methionine concentration within the cell, interfering in protein synthesis [74,75].

Another transporter, histidine permease, also decreased in abundance in *C. neoformans* cells after exposure to *Mo*-CBP_3_-PepII (Supplementary Table S1). As happens to methionine, histidine is a proteinogenic essential amino acid that has to be collected by cells from the environment [76,77,78,79]. However, otherwise than methionine permease, histidine permease functions go beyond histidine transport. In fungi, histidine permeases are essential to keep fungi safe, virulent, vigorous, and normal morphogenesis and development [78,79]. The absence of this protein in *C. neoformans* cells treated with *Mo*-CBP_3_-PepII suggests that all processes developed by it are compromised, making it hard for the fungus to overcome stresses imposed by the peptide.

Another protein that decreased in abundance in *C. neoformans* cells exposure to *Mo*-CBP_3_-PepII was the oligomycin resistance ATP-dependent permease YOR1 (Table 1). Oligomycin is a molecule produced by bacteria from the *Streptomyces* genus and is used as an antibiotic. Oligomycin binds to ATP synthase inhibiting ATP synthesis [80,81,82]. Over the years, pathogenic yeasts have developed resistance to oligomycin by producing oligomycin resistance ATP-dependent permease YOR1 [81]. Thus, the reduction in the abundance of oligomycin resistance ATP-dependent permease YOR1 suggests that *C. neoformans* cells became susceptible to oligomycin after treatment with *Mo*-CBP_3_-PepII.

A similar result was recently published by Branco et al. [22], revealing that the treatment of *Klebsiella pneumoniae* cells with the *Mo*-CBP_3_-PepI synthetic peptide also induces the reduction in three multidrug resistance proteins such as multidrug resistance protein MdtN, UPF0194 membrane protein YbhG, and multidrug resistance protein EmrK. This result suggests *Mo*-CBP_3_-PepI increases the susceptibility of *K. pneumoniae* to drugs. The same result was found in *C. neoformans* after treatment with *Mo*-CBP_3_-PepII.

The BNI4 protein with the highest fold-change of 66.18 shows its significant accumulation in *C. neoformans* cells after treatment with *Mo*-CBP_3_-PepII (Table 1). The BNI4 protein is involved in the recruitment of chitin synthase to produce the chitin to be incorporated in the cell wall of the new buddy [83,84]. Yeasts divide by cytokinesis, a process where one cell divides itself in two [85]. During this process, the cell wall needs to be produced for two cells; for this process, chitin synthase has to be recruited by the BNI4 protein [83,84,85].

We have one hypothesis for this higher level of BNI4 protein. *Mo*-CB_3_-PepII is a synthetic peptide designed from the sequence of a chitin-binding protein from *Moringa oleifera* seeds, Mo-CBP_3_ [11]. Lima et al. [86] proved that *Mo*-CBP_3_-PepII is a chitin-binding peptide and causes damage to the cell wall of *Candida albicans* by interacting with chitin. Scanning electron microscopy reported by Aguiar et al. [10] was corroborated by AFM analysis (Figure 5), which was revealed here strongly suggests that *Mo*-CBP_3_-PepII also in *C. neoformans* cell wall. As such, we hypothesize that *Mo*-CBP_3_-PepII might interact with chitin and thus interfere with producing cell walls for new buds. To cope with this insult, *C. neoformans* cells raised the levels of BNI4 as much as possible to recruit chitin synthase to produce cell wall for new buds.

In contrast to BNI4, coronin is the protein with the lowest fold-change value of 0.04 (Table 1) and thus decreases in abundance in *C. neoformans* cells treated with *Mo*-CBP_3_-PepII. Coronin is a protein critical for cell structure because it interacts with actin filaments and microtubules, promoting cellular processes such as remodeling the cell cytoskeleton, cell motility, endocytosis, and phagocytosis [87,88,89]. In yeasts, Cai et al. [89] reported that coronin protects actin filaments from depolymerization keeping the cytoskeleton functional. In contrast, in coronin-mutant yeasts, the function of actin filaments and cytoskeleton are compromised [89]. Those coronin-mutant yeasts lost the ability to remodel the cytoskeleton as well as have other cytoskeleton-dependent functions compromised. Here, the dramatic reduction in the levels of coronin in peptide-treated *C. neoformans* cells suggests that *Mo*-CBP_3_-PepII is affecting the well-function of the cytoskeleton and inhibiting critical processes to cell life. Recently, Chen et al. [23] reported alteration in the transcriptome of *C. neoformans* with the synthetic peptide Sparamosin_26–54_. The authors revealed a reduction in the expression of four genes involved in cell wall metabolism by RNA-seq technology [23].

#### 2.7.8. Energetic Metabolism-Related Proteins

The most exciting proteins were identified exclusively in *C. neoformans* cells treated com *Mo*-CBP_3_-PepII (Supplementary Table S2). The first protein found was Cyt c mitochondrial import. The Cyt c mitochondrial import is a protein that imports the cytoplasmic Cyt c to the mitochondrial membrane [90,91,92,93]. The exclusive identification of this protein in *C. neoformans* cells treated with *Mo*-CBP3-PepII is very exciting. As pointed out above, *Mo*-CBP_3_-PepII induced the decoupling of Cyt c from the mitochondrial membrane (Figure 4C). Therefore, the exclusive identification of Cyt c mitochondrial import in *C. neoformans* treated cells indicates strongly suggests the cell is trying to respond to the stresses caused by peptide either by producing new Cyt c molecules or even recoupling the molecules released by the peptide.

Another protein exclusively identified in treated cells was alcohol dehydrogenase 4 (Supplementary Table S2). Alcohol dehydrogenase functions in the alcoholic fermentation of yeasts [94,95]. In alcohol fermentation, the pyruvate is produced in glycolysis. After glycolysis, pyruvate is driven to a two-reaction reaction, where it is converted into ethanol in a step catalyzed by alcohol dehydrogenase [94,95]. The point in the alcoholic formation is that, in the reduction of pyruvate to ethanol, the NADH cytosolic produced in the glycolysis is converted into NAD^+,^, raising the ratio NAD^+^/NADH and thus favoring the glycolysis [94,95].

The point is, in the *C. neoformans* treated cells, a reduction in the activity of LDH induced by *Mo*-CBP_3_-PepII (Figure 4A). LDH recovered the NAD^+^ by lactic fermentation, keeping high the NAD^+^/NADH ratio to favor glycolysis [32]. So, it is feasible to suggest that the high level of alcohol dehydrogenase is an attempt of *C. neoformans* cells to compensate for the inhibition of LDH by *Mo*-CBP_3_-PepII and stand high the NAD^+^/NADH ratio to favor glycolysis.

#### 2.7.9. Pathogenicity-Related Proteins

In this group, one protein exclusively detected in control cells and absent in the treated deserves attention, the Subtilisin-like protease 6 (Supplementary Table S1 and S2). The Subtilisin-like proteases are enzymes in fungi autophagy and fungal virulence, enhancing invasion and colonization [96,97]. The absence of Subtilisin-like protease 6 after treatment with *Mo*-CBP_3_-PepII (Supplementary Table S2) indicates the *C. neoformans* cells stay with a limited capacity to cause infection.

It is essential to notice that recent studies showed that *Mo*-CBP_3_-PepII is a safe molecule presenting no toxicity to human erythrocytes, MRC-5, HaCAT, L292 human cell lines, and zebrafish embryos [11,98]. These studies reinforce the potential of *Mo*-CBP_3_-PepII for developing new strategies to combat the infection of *C. neoformans*.

## 3. Conclusions

The results presented here showed that *Mo*-CBP_3_-PepII displays its anticryptococcal activity by multiple mechanisms of action affecting several cellular processes essential for cell life, such as development, pathogenesis, cell division, and metabolism. Acting on cells in multiple ways, *Mo*-CBP_3_-PepII makes difficult the development of resistance by *C. neoformans*. Additionally, our results suggest that *Mo*-CBP_3_-PepII could act as an adjuvant for drugs that are becoming useless. Therefore, it is feasible to suggest that *Mo*-CBP_3_-PepII is a potential molecule to be employed alone or in combination with other drugs to develop new treatments against *C. neoformans*.

## 4. Materials and Methods

### 4.1. Fungal Strains, Chemicals, and Synthetic Peptides

*Cryptococcus neoformans* (ATCC 32045) was obtained from the Department of Biochemistry and Molecular Biology at the Federal University of Ceará (UFC), Fortaleza, Brazil. All the chemicals used in the experiments were obtained from Sigma Aldrich (São Paulo, SP, Brazil). The *Mo*-CBP_3_-PepII (NIQPPCRCC) synthetic peptide was chemically synthesized by the company ChemPeptide (Shanghai, China) with a purity of >98%.

### 4.2. Antifungal Assay

The antifungal assay was performed according to Aguiar et al. [10]. The cryptococcal cells were cultivated in yeast potato dextrose (YPD) agar for 10 days. Then cells were collected and transferred to a liquid YPD medium. The concentration of *Mo*-CBP_3_-PepII was the MIC_50_ (25 µg mL^−1^) defined by Aguiar et al. [10]. This MIC_50_ concentration was used in all experiments performed in this study. Therefore, 25 µL of *C. neoformans* cells (10^6^ cells mL^−1^) and 25 µL of *Mo*-CBP_3_-PepII (25 µg mL^−1^) were incubated for 24 h at 30 °C before each assay. The solution of 5% DMSO in 0.15 M NaCl (DMSO-NaCl) was the control used.

### 4.3. Detection of Mo-CBP_3_-PepII-Induced Overproduction of ROS

A fluorescence microscopy assay was performed to evaluate the *Mo*-CBP_3_-PepII-induced H_2_O_2_ overproduction, as described previously by Dias et al. [99]. After the antifungal assay (Section 4.2), the samples were washed with 0.15 M NaCl and centrifugated (5000× *g* 5 min at 4 °C) three times and then were incubated with 9 µL of 2′,7′ dichlorofluorescein diacetate (DCFH-DA, Sigma, St. Louis, MI, USA) for 30 min in the dark at 22 ± 2 °C. Next, the samples were washed and centrifuged as mentioned, transferred to slides, and observed with a fluorescence microscope (Olympus System BX 41, Tokyo, Japan) with an excitation wavelength of 535 nm and 370 emission wavelength of 617 nm.

Additionally, the qualitative of anion superoxide was performed according to Choi et al. [100]. The antifungal assay was conducted as above. Then *C. neoformans* cells were washed, recovered in 0.15 M of NaCl, and incubated with 100 µM nitroblue tetrazolium chloride (NBT) (Sigma Aldrich, St. Louis, MI, USA) for 2 h at 22 ± 2 °C. Then, cells were washed to remove the excess NBT and visualized in a light microscope (Olympus System BX 41, Tokyo, Japan).

The experiments to evaluate the anticryptococcal activity in the presence of the antioxidant agent ascorbic were conducted following the methodology described by Neto et al. [28]. The experiments to perform the H_2_O_2_ overaccumulation and pore formation in ascorbic acid at 10 mM were performed as described by Aguiar et al. [21].

### 4.4. Protein Extraction from C. neoformans Cells

The extraction of proteins from *C. neoformans* cells was conducted following Branco et al. [22]. After the antifungal assay, the samples were washed three times to remove the media with 50 mM Na^+^-acetate pH 5.2 and centrifugated at 12,000× *g* for 15 min at 4 °C. Then, samples were resuspended in 200 µL of extraction buffer and frozen at −20 °C for 24 h. After that, the samples were submitted to sonication for 30 min to break the cell wall and plasmatic membrane and centrifuged again, and the supernatant was collected. The Bradford assay [101] was performed to determine the protein concentration using bovine serum albumin (BSA). The extracted proteins were used for enzymatic assay and proteomic analysis.

### 4.5. Activity Redox System Enzymes

#### 4.5.1. Ascorbate Peroxidase (APX) Activity

To evaluate the APX activity, the methodology was performed according to Souza et al. [102]. In tubes, 100 μL of the treated or control samples were mixed with 800 μL of 50 mM K^+^-phosphate buffer, pH 6.0, containing 0.5 mM of L-ascorbic acid and incubated at 30 °C for 10 min. Then, 100 μL of 2 mM H_2_O_2_ was added to start the reaction. The reaction was tracked every 10 s until 1 min in the spectrophotometer (Biochron, Libra 394 S12) at the wavelength 290 nm. APX activity unity (AU) was expressed as the reduction in the absorbance by 0.01 at 290 nm, indicating the use of ascorbate to remove H_2_O_2_ by milligram of the protein (UA mgP^−1^).

#### 4.5.2. Catalase (CAT) Activity

Following Souza et al. [101], 200 μL of proteins extracted from *Mo*-CBP_3_-PepII-treated and non-treated *C. neoformans* cells were incubated with 700 μL 50 mM K^+^-phosphate buffer pH 7.0 at 30 °C for 10 min. Next, 100 μL of H_2_O_2_ were added to start the reaction. The reaction medium was transferred to a quartz cuvette (1 cm^−1^), and the absorbance was measured in a spectrophotometer (Biochron, Libra 394 S12). The decrease in absorbance at 240 nm was observed at intervals of 10 s to 1 min. CAT activity unity (AU) was expressed as the reduction in the absorbance by 0.01 at 240 nm, indicating the use of ascorbate to remove H_2_O_2_ by milligram of the protein (UA mgP^−1^).

#### 4.5.3. Superoxide Dismutase (SOD) Activity

SOD activity was performed following the methodology described by Souza et al. [102]. In a flat-bottom 96 well-plate, were mixed 10 μL of 1 M K^+^-phosphate buffer pH 7.8, 20 μL 1 mM 2,2′,2″,2‴- ethylenediaminetetraacetic acid (EDTA), 10 μL of 0.25% Triton X-100, 20 μL of 130 mM L-Methionine, 100 μL of protein extract from *C. neoformans* cells in the presence and absence of *Mo*-CBP_3_-PepII (MIC_50_), 20 μL 100 mM of riboflavin, and 20 μL of 700 μM of BNT. The mixture was homogenized and kept in the dark for 5 min. After that, the plate was exposed to a 32 W fluorescent light, and the absorbances were measured in 1 min intervals up to 5 min at 630 nm in a microplate reader (Epoch, Biotek, Santa Clara, CA, USA). Blanks consisted of all reagents used without protein extracts; it was replaced with deionized water. The SOD was expressed in activity units per mg of protein (AU mgP^−1^). One unit of SOD activity (1 AU) corresponded to the amount of the sample needed to inhibit the photoreduction of NBT by 50%.

### 4.6. Ergosterol Inhibition Synthesis

The inhibition of ergosterol biosynthesis was evaluated according to Neto et al. [28]. The *C. neoformans* cells were cultivated in the presence of *Mo*-CBP_3_-PepII (MIC_50_), 5% DMSO, and Itraconazole (ITR) (1000 µg mL^−1^) for 24 h at 30 °C. Then, the cells were centrifugated at 3000× *g* for 5 min. Subsequently, the pellet was dried and weighed. Two mL of 25% alcoholic sodium hydroxide solution (*m*/*v*) were added to each pellet and strongly vortexed for 1 min. To extract the sterol, 4 mL of sterile 75% *n*-hexane were added and vortexed for 3 min. Then, 400 μL of pure ethanol were added to 200 μL sterol extract. Next, the samples were transferred to a quartz cuvette (1 cm), and the absorbance was measured in a spectrophotometer (Biochron, Libra 394 S12) at 230 nm and 282 nm. Ergosterol content was calculated based on three equations:

% ergosterol + 24 (28) [DHE = (Abs282/290) × F]/pellet weight(1)

% 23 (28) DHE = [(Abs230/518) × F]/pellet weight(2)

% ergosterol = % ergosterol + 24 (28) DHE − % 24 (28) DHE(3)

The part of the equation that is 24 (28) DHE refers to dehydroergosterol, where the absorbance is similar to ergosterol at 282 nm. F, in both equations, represents the factor of dilution of ethanol.

### 4.7. Lactate Dehydrogenase (LDH) Activity

The LDH Liquiform™ kit (Labtest Diagnóstica, Lagoa Santa, MG, Brazil) was measured according to the manufacturer’s instructions.

### 4.8. Cyt c Release

The assay was performed according to Neto et al. [28]. The cells were incubated with *Mo*-CBP_3_-PepII (MIC_50_), H_2_O_2_ (10 mM), and DMSO-NaCl for 24 h at 30 °C. Then, 100 µL of buffer 50 mM Tris-HCl pH 7.5 containing 2 mM EDTA, 1 mM phenylmethylsulphonyl fluoride, and 6% glucose were added to the cell suspension and homogenized. Subsequently, the samples were centrifuged at 2000× *g* at 4 °C for 10 min, and the supernatant was collected and added into microtubes. The pellet was washed with the buffer 50 mM Tris pH 7.5 containing 2 mM EDTA and centrifugated (6000× *g* for 30 s). The supernatant was discarded, and the mitochondria were suspended in 100 µL of the same buffer. Next, the cytosolic and mitochondrial suspensions were treated with 30 mM of ascorbic acid for 5 min at 30 °C. Then, the optical density of the obtained solution was measured using a microtiter plate reader (Epoch, Biotek, Santa Clara, CA, USA) at 550 nm.

### 4.9. Atomic Force Microscopy (AFM)

After the antifungal assay, the cells were prepared for AFM analysis following the methodology described by Souza et al. [103]. The samples were washed three times with sterile water and centrifugated at 5000× *g* for 5 min at 4 °C. Then, 10 μL of the cells were transferred to a freshly washed glass surface previously treated with (*v*/*v*) poly-L-lysine and incubated at room temperature (22 ± 2°C) for 30 min. All samples were analyzed on Multimode 8 (Bruker, Santa Barbara, CA, USA). The probes used were SNL (Bruker, Billerica, MA, USA) with 0.06 N/m nominal spring constant, a resonance frequency of 320 kHz, and a nominal tip radius of 8 nm.

### 4.10. Gel-Free Proteomic Analysis

For proteomic analysis, proteins were extracted as described in Section 4.4. Subsequently, proteins from the control and treated cells were treated with 10 mM DTT and incubated for 1 h at 37 °C in the dark to reduce the proteins. Next, 15 mM iodoacetamide were added and incubated for 30 min in a dark room to alkylate the reduced proteins. Then, the proteins were digested using trypsin gold (Promega, Madison, WI, USA) to a final concentration of 1:20 (*w*/*w*) as described by manufacturers for 16 h at 37 °C. After that, the samples were dried in a speed vacuum (Eppendorf, Hamburg, Germany) for 3 h and analyzed by an ESI-QUAD-TOF mass spectrometer.

### 4.11. Protein Identification

The protein identification was performed following Branco et al. [22]. Tandem mass spectra were exported as .pkl files and loaded in the MASCOT MS/MS ions search from MATRIX SCIENCE (https://www.matrixscience.com/cgi/search_form.pl?FORMVER=2&SEARCH=MIS, accessed on 10 November 2022) against UP2311_*S*_*cerevisiae* (protein database), UP219602_*F*_*oxysporum* (protein database), and SwissProt databases (protein database). The search was conducted by the following parameters: fixed modifications to Carbamidomethyl (C), variable modifications to Oxidation (O); the peptide charge was set to 2+, 3+, and 4+; and the instrument was set to ESI-QUAD-TOF. The proteins identified were searched for in UNIPROT and separated into 3 sets: (1) unique from control for those only identified in control samples, (2) unique from the cells treated with *Mo*-CBP_3_-PepII for those only identified in treated samples, and (3) *Mo*-CBP_3_-PepII x control overlapping proteins.

The proteins with a fold-change value ≥1.5 (*p* < 0.05, Tukey’s test) were up-accumulated (increased the abundance), and proteins with a fold-change value ≤0.5 (*p* < 0.05, Tukey’s test) were down-accumulated (decreased the abundance) and considered for comparisons. Proteins with a fold-change value between 0.5 and 1.5 were considered not to have changed. For each protein, its corresponding FASTA file was downloaded. The blast2go program (https://www.blast2go.com/, accessed on 25 November 2022) was used to categorize the proteins detected by Gene Ontology (GO) annotation according to molecular function, biological Activity, and subcellular location.

### 4.12. Statistical Analysis

All experiments were performed in triplicates. The statistical analyses were performed using GraphPad Prism (version 5.01) for Microsoft Windows. All data obtained in the assays were compared using the one-way analysis of variance (ANOVA), followed by the Tukey test (*p* < 0.05).

## Figures and Tables

**Table 1:** Overlapping proteins identified in control and treated cells of *C. neoformans* by LC-ESI-MS/MS analysis.

Protein Identification	Uniprot/NCBI Code	Reference Organisms	Cellular Component	Fold-Change Treated/Control Cells
DNA and RNA Binding				
Transcriptional activator SPT7	P35177	*Saccharomyces cerevisiae*	Nucleus	0.695080677
Sister chromatid cohesion protein 2	Q04002	*Saccharomyces cerevisiae*	Chromosome	0.82634375
Protein cft1	A2R919	*Aspergillus niger*	Nucleus	0.82634375
ATP-dependent RNA helicase DBP4	Q6CRF4	*Kluyveromyces lactis*	Nucleus	1.010452416
Transcription activator of gluconeogenesis ERT1	Q754V2	*Ashbya gossypii*	Nucleus	0.772658613
Signal recognition particle 54 kDa protein homolog	Q00179	*Aspergillus niger*	Endoplasmic reticulum	0.677367871
Hydrolase				
Cyanide hydratase	P9WEU5	*Stereum hirsutum*	Cytoplasm	0.862324582
Serine/threonine-protein phosphatase 4 catalytic subunit	Q74ZR2	*Ashbya gossypii*	Nucleus	0.977511571
Ligase				
Carbamoyl-phosphate synthase arginine-specific large chain	P46056	*Cutaneotrichosporon cutaneum*	Cytoplasm	0.070970139
Multifunctional enzyme				
Nonribosomal peptide synthase agiA	B8NY88	*Aspergillus flavus*	Endoplasmic reticulum	0.609201703
Oxidoreductase				
D-2-hydroxyglutarate--pyruvate transhydrogenase DLD3	P39976	*Saccharomyces cerevisiae*	Cytoplasm	1.992460047
3-isopropylmalate dehydrogenase A	P87256	*Aspergillus niger*	Cytoplasm	1.782962796
Acyl-coenzyme A oxidase	Q6FY63	*Candida glabrata*	Peroxisome	1.03315885
Alcohol dehydrogenase 2	P54202	*Emericella nidulans*	Cytoplasm	0.609201703
Protein Binding				
Rab proteins geranylgeranyltransferase component A	P32864	*Saccharomyces cerevisiae*	Nucleus	0.561076893
Pre-mRNA-splicing factor ATP-dependent RNA helicase PRP22	P24384	*Saccharomyces cerevisiae*	Unknown	0.815963494
Autophagy-related protein 11	Q5AMN3	*Candida albicans*	Vacuole	1.320278149
ADP-ribosylation factor GTPase-activating protein effector protein 2	P40529	*Saccharomyces cerevisiae*	Cytoplasm/nucleus	0.909705632
Transferase				
E3 ubiquitin-protein ligase TOM1	Q03280	*Saccharomyces cerevisiae*	Nucleus	0.968391082
Atypical kinase COQ8. mitochondrial	P27697	*Saccharomyces cerevisiae*	Mitochondrion	1.865633075
Threonylcarbamoyl-AMP synthase	P32579	*Saccharomyces cerevisiae*	Chromosome	8.393193801
Checkpoint serine/threonine-protein kinase BUB1	P41695	*Saccharomyces cerevisiae*	Nucleus	8.819735039
Ribosomal RNA small subunit methyltransferase NEP1	Q06287	*Saccharomyces cerevisiae*	Nucleus	0.977511571
GST N-terminal domain-containing protein	A0A2H3HTS3	*Fusarium oxysporum f. sp.*	Nucleus	1.865633075
(2E.6E)-farnesyl diphosphate synthase	A0A2H3HBS6	*Fusarium oxysporum f. sp.*	Cytoplasm/nucleus	11.124331
Spindle assembly checkpoint kinase	Q755C4	*Ashbya gossypii*	Chromosome	5.871423002
O-methyltransferase cicE	A0A1U8QH20	*Emericella nidulans*	cytoplasm	1.08068077
ATP-dependent 6-phosphofructokinase 1	Q9HGZ1	*Aspergillus oryzae*	cytoplasm	0.894731439
Homoserine kinase	Q92209	*Candida albicans*	cytoplasm	0.791267101
Transport or Structural Activity				
Low-affinity methionine permease	P38734	*Saccharomyces cerevisiae*	Membrane	0.213834043
Oligomycin resistance ATP-dependent permease YOR1	P53049	*Saccharomyces cerevisiae*	Membrane	0.147024894
Protein BNI4	P53858	*Saccharomyces cerevisiae*	Membrane	66.18226428
Coronin	A0A2H3H7T3	*Fusarium oxysporum f. sp.*	Cytoplasm	0.043680452
Actin cytoskeleton-regulatory complex protein PAN1	Q1DQC1	*Coccidioides immitis*	Membrane	0.947544705
Unknown				
CIA30 domain-containing protein	A0A2H3GUN4	*Fusarium oxysporum f. sp.*	Mitochondrion	0.274170461
Velvet domain-containing protein	A0A2H3H7M3	*Fusarium oxysporum f. sp.*	Nucleus	0.761358738
Bud site selection protein RAX1	Q08760	*Saccharomyces cerevisiae*	Membrane	0.274170461
Required for respiratory growth protein 8. mitochondrial	Q6CQQ8	*Kluyveromyces lactis*	Mitochondrion	1.398398098
Proteasome subunit alpha type-1	P21243	*Saccharomyces cerevisiae*	Cytoplasm	1.320399815
Imizoquin biosynthesis cluster protein A	B8NI18	*Aspergillus flavus*	Cytoplasm	1.108964381
Protein mago nashi homolog	O43037	*Schizosaccharomyces pombe*	Nucleus	0.249951752

## Data Availability

The data supporting this study’s findings are available upon request from the corresponding author.

## Supplementary Materials

The following supporting information can be downloaded at: https://www.mdpi.com/article/10.3390/antibiotics12020378/s1, Table S1: Unique proteins identified in the DMSO group by ESI-LC-MS/MS; Table S2: Unique proteins identified in the treated group by ESI-LC-MS/MS.
