# Supplementary material for: No Chance to Survive: Mo-CBP3-PepII Synthetic Peptide Acts on Cryptococcus neoformans by Multiple Mechanisms of Action
Source: Antibiotics (Basel). 2023 Feb 12;12(2):378. doi: 10.3390/antibiotics12020378 (PMC9952340; doi:10.3390/antibiotics12020378)
Supplement: Supplementary file 1 [file antibiotics-12-00378-s001.zip › antibiotics-2201833-supplementary.pdf]

Supplementary Table S1 – Unique proteins identified in the DMSO group by ESI-LC-MS/MS

Protein Name	ID (Uniprot)	Organism Reference	Cellular Compartment
Transport			
Succinate dehydrogenase [ubiquinone] cytochrome b subunit, mitochondrial	P33421	*Saccharomyces cerevisiae*	Mitochondrion inner membrane
Anion/proton exchange transporter GEF1	P37020	*Saccharomyces cerevisiae*	Golgi apparatus membrane
Sodium transport ATPase 1	P13587	*Saccharomyces cerevisiae*	Cell membrane
Vacuolar protein sorting-associated protein 16	Q96QK1	*Saccharomyces cerevisiae*	Vacuole
V-type proton ATPase catalytic subunit A	P32563	*Saccharomyces cerevisiae*	Vacuole membrane
Protein CASP	P34237	*Saccharomyces cerevisiae*	Golgi apparatus membrane
Exportin-1	P30822	*Saccharomyces cerevisiae*	Cytoplasm
Na(+)/H(+) antiporter	B3LT99	*Saccharomyces cerevisiae*	Membrane
ATP-dependent permease AUS1	Q08409	*Saccharomyces cerevisiae*	Membrane
Histidine permease	P06775	*Saccharomyces cerevisiae*	Membrane
ATPase GET3	Q12154	*Saccharomyces cerevisiae*	Cytoplasm
Protein SLY1	P22213	*Saccharomyces cerevisiae*	Cytoplasm
Ferric reductase transmembrane component 5	Q12473	*Saccharomyces cerevisiae*	Cytoplasm

Vacuolar membrane-associated protein IML1	P47170	*Saccharomyces cerevisiae*	Cytoplasm
Magnesium transporter	W9KFF0	*Fusarium oxysporum*	Mitochondrion inner membrane
RanBD1 domain-containing protein	A0A0J9UT92	*Fusarium oxysporum*	Nucleus
Bifunctional cytochrome P450/ NADPH--P450 reductase	Q9Y8G7	*Fusarium oxysporum*	Cytoplasm
GPI inositol-deacylase	Q5AYC8	*Emericella nidulans*	Endoplasmic reticulum membrane
Serine/threonine-protein kinase KIN82	P25341	*Saccharomyces cerevisiae*	Cytoplasm
Biogenesis of lysosome-related organelles complex 1 subunit	Q75AC2	*Ashbya gossypii*	Cytoplasmic vesicle
Mitochondrial inner membrane magnesium transporter	Q4I298	*Gibberella zeae*	Mitochondrion inner membrane
Nuclear distribution protein nudE homolog 1	Q4P0N6	*Ustilago maydis*	Cytoplasm
Pheromone-regulated membrane protein 9	P39551	*Saccharomyces cerevisiae*	Membrane
Nucleoporin NUP56	G0S8I1	*Chaetomium thermophilum*	Nuclear pore complex
Polyamine transporter 2	P53283	*Saccharomyces cerevisiae*	Membrane
Nuclear protein localization protein 4	Q6BRJ9	*Debaryomyces hansenii*	Cytoplasm
Vacuolar protein sorting/targeting protein 10	Q5AS50	*Emericella nidulans*	Golgi apparatus
Mechanosensitive ion channel protein Msy1	O74839	*Schizosaccharomyces pombe*	Endoplasmic reticulum membrane

COPII coat assembly protein SEC16	Q6CEV2	*Yarrowia lipolytica*	Endoplasmic reticulum membrane
Mitochondrial glycine transporter	A3M019	*Scheffersomyces stipites*	Mitochondrion inner membrane
GTP-binding protein gtr1	O74824	*Schizosaccharomyces pombe*	Vacuole membrane
V-type proton ATPase subunit a	Q01290	*Neurospora crassa*	Vacuole membrane
Phosphate metabolism protein 7	Q12252	*Saccharomyces cerevisiae*	Membrane
Cell Function and Structure			
Nuclear migration protein NUM1	Q00402	*Saccharomyces cerevisiae*	Bud tip
Peroxisomal ATPase PEX6	P33760	*Saccharomyces cerevisiae*	Peroxisome membrane
Killer toxin-resistance protein 5	P22023	*Saccharomyces cerevisiae*	Endoplasmic reticulum
Condensin complex subunit 3	Q06680	*Saccharomyces cerevisiae*	Nucleus
Altered inheritance of mitochondria protein 20	P40451	*Saccharomyces cerevisiae*	Vacuole membrane
GTPase-interacting component 2	P38785	*Saccharomyces cerevisiae*	Bud neck
Non-SCF-type F-box protein ROY1	Q04847	*Saccharomyces cerevisiae*	Cytoplasm
MICOS complex subunit MIC19	P43594	*Saccharomyces cerevisiae*	Mitochondrion inner membrane
Nucleoporin ASM4	Q05166	*Saccharomyces cerevisiae*	Nucleus
HMG2-induced ER-remodeling protein 1	Q12276	*Saccharomyces cerevisiae*	Cytoplasm
Morphogenesis-related protein MSB1	P21339	*Saccharomyces cerevisiae*	Cellular bud neck

1,3-beta-glucan synthase component FKS1	P38631	*Saccharomyces cerevisiae*	Mitochondrion
Nuclear fusion protein BIK1	P11709	*Saccharomyces cerevisiae*	Cytoplasm
Cell division control protein 48	P25694	*Saccharomyces cerevisiae*	Endoplasmic reticulum
Midasin	A0A2H3T3C5	*Fusarium oxysporum*	Nucleus
Dynein heavy chain, cytoplasmic	A0A0D2XQR5	*Fusarium oxysporum*	Cytoplasm
Dynamin-type G domain-containing protein	A0A2H3STJ2	*Fusarium oxysporum*	Nucleus/Cytoplasm
Spindle pole body component	A0A0D2XX36	*Fusarium oxysporum*	Cytoplasm
Protein sts5	O74454	*Saccharomyces pombe*	Cytoplasm
Kinesin-like protein bimC	P17120	*Emericella nidulans*	Cytoplasm
Myosin-1	A7TDZ8	*Vanderwaltozyma polyspora*	Cytoplasm
Actin cytoskeleton-regulatory complex protein E	L7IIY8	*Magnaporthe oryzae*	Membrane
Vacuolar protein sorting-associated protein 3	P23643	*Saccharomyces cerevisiae*	Cytoplasm
Chitin deacetylase 3	P82476	*Cryptococcus neoformans*	Membrane
Actin-regulating kinase PRK1	Q5A961	*Candida albicans*	Cytoplasm
Karyogamy meiotic segregation protein 1	P87245	*Schizosaccharomyces pombe*	Cytoplasm
Target of rapamycin complex	O74184	*Schizosaccharomyces pombe*	Cytoplasm

subunit wat1			
ATP-dependent DNA helicase CHL1	Q750G3	*Ashbya gossypii*	Nucleus
Separin	P18296	*Schizosaccharomyces pombe*	Nucleus
Energetic metabolism			
ATP synthase subunit d, mitochondrial	P30902	*Saccharomyces cerevisiae*	Mitochondrion inner membrane
Glyceraldehyde-3-phosphate dehydrogenase 2	P00358	*Saccharomyces cerevisiae*	Cytoplasm
6-phosphogluconate dehydrogenase, decarboxylating 1	P38720	*Saccharomyces cerevisiae*	Cytoplasm
FAD-linked oxidoreductase chry5	I1S3L1	*Gibberella zeae*	Mitochondrion
D-2-hydroxyglutarate—pyruvate transhydrogenase DLD3	P39976	*Saccharomyces cerevisiae*	Cytoplasm
NADH-ubiquinone oxidoreductase 49 kDa subunit, mitochondrial	P22142	*Neurospora crassa*	Mitochondrion inner membrane
Cytochrome c oxidase assembly protein COX16, mitochondrial	Q4I8P5	*Gibberella zeae*	Mitochondrion inner membrane
Protein Folding and metabolism			
26S proteasome regulatory subunit RPN1	P38764	*Saccharomyces cerevisiae*	Cytoplasm
Elongation factor 2	P32324	*Saccharomyces cerevisiae*	Cytoplasm
26S proteasome regulatory subunit RPN5	Q12250	*Saccharomyces cerevisiae*	Proteasome

Tyrosine-protein phosphatase 3	P40048	*Saccharomyces cerevisiae*	Cytoplasm
U3 small nucleolar RNA-associated protein MPP10	P47083	*Saccharomyces cerevisiae*	Nucleus
ERAD-associated E3 ubiquitin-protein ligase component HRD3	P47083	*Saccharomyces cerevisiae*	Endoplasmatic reticulum
Chromosome instability protein 1	P40987	*Saccharomyces cerevisiae*	Endoplasmatic reticulum
Kexin	P13134	*Saccharomyces cerevisiae*	Golgi apparatus
Peptide-N(4)-(N-acetyl-beta-glucosaminyl) asparagine amidase	Q02890	*Saccharomyces cerevisiae*	Cytoplasm
Mitochondrial chaperone BCS1	P32839	*Saccharomyces cerevisiae*	Cytoplasm
Serine/threonine-protein kinase AKL1	P38080	*Saccharomyces cerevisiae*	Cellular bud neck
ER membrane protein complex subunit 6	P25574	*Saccharomyces cerevisiae*	Endoplasmic reticulum membrane
Protein CSF1	Q12150	*Saccharomyces cerevisiae*	Membrane
Assembly chaperone of RPL4	Q03771	*Saccharomyces cerevisiae*	Cytoplasm
Protein TMA108	P40462	*Saccharomyces cerevisiae*	Cytoplasm
Protein ASI1	P53895	*Saccharomyces cerevisiae*	Nucleus inner membrane
F-box protein YLR352W	Q06479	*Saccharomyces cerevisiae*	SCF ubiquitin ligase complex
Peptide hydrolase	X0CEB5	*Fusarium oxysporum*	Mitochondrion
AAA domain-containing protein	A0A0D2Y4N3	*Fusarium oxysporum*	Nucleus

TYR_PHOSPHATASE_2 domain-containing protein	A0A6M7ZMH6	*Fusarium oxysporum*	Cytoplasm
Peptidase_M14 domain-containing protein	W9KWL0	*Fusarium oxysporum*	Extracellular region
Protein kinase domain-containing protein	A0A0D2XQE2	*Fusarium oxysporum*	Nucleus
UBA_e1_C domain-containing protein	F9FV89	*Fusarium oxysporum*	Cytoplasm
Clustered mitochondria protein homolog	A0A2H3TIH9	*Fusarium oxysporum*	Cytoplasm
GPI ethanolamine phosphate transferase 2	A0A6M7ZB90	*Fusarium oxysporum*	Endoplasmic reticulum membrane
5-aminolevulinate synthase	A0A0D2XE20	*Fusarium oxysporum*	Mitochondrion
Eukaryotic translation initiation factor 3 subunit M	Q4P0P0	*Ustilago maydis*	Cytoplasm
Protein rot1	A6S3W1	*Botryotinia fuckeliana*	Endoplasmic reticulum membrane
Protein SUE1, mitochondrial	Q06524	*Saccharomyces cerevisiae*	Mitochondrion
Neutral protease 2 homolog MGG_10927	E3QWD3	*Colletotrichum graminicola*	Secreted
Uba3-binding protein but2	P87167	*Schizosaccharomyces pombe*	Nucleus
Ubiquinone biosynthesis protein coq4, mitochondrial	B6QTE1	*Talaromyces marneffei*	Mitochondrion inner membrane

Eukaryotic translation initiation factor 3 subunit K	Q1E6D3	*Coccidioides immitis*	Cytoplasm
7-dimethylallyltryptophan synthase	Q4WYG3	*Neosartorya fumigate*	Cytoplasm
N-(5-amino-5-carboxypentanoyl)-L-cysteinyl-D-valine synthase	P27742	*Emericella nidulans*	Cytoplasm
Methionine aminopeptidase 2-2	B6QD96	*Talaromyces marneffei*	Cytoplasm
Carboxypeptidase Y homolog A	C5P212	*Coccidioides posadasii*	Vacuole
DNA and RNA binding			
tRNA (guanosine(18)-2'-O)-methyltransferase	P38238	*Saccharomyces cerevisiae*	Cytoplasm
Transposon Ty3-G Gag-Pol polyprotein	Q12173	*Saccharomyces cerevisiae*	Cytoplasm
Transcription elongation factor SPT5	P27692	*Saccharomyces cerevisiae*	Nucleus
54S ribosomal protein L49, mitochondrial	P40858	*Saccharomyces cerevisiae*	Mitochondrion
Nuclear localization sequence-binding protein	P27476	*Saccharomyces cerevisiae*	Nucleus
Mediator of RNA polymerase II transcription subunit 15	P32569	*Saccharomyces cerevisiae*	Nucleus
RNA cytidine acetyltransferase	P53914	*Saccharomyces cerevisiae*	Nucleus
Y' element ATP-dependent helicase protein 1 copy 1	O13559	*Saccharomyces cerevisiae*	Cytoplasm
Pre-mRNA-splicing factor ATP-dependent RNA helicase-like protein	P20095	*Saccharomyces cerevisiae*	Nucleus
Chromosome transmission fidelity	P49956	*Saccharomyces cerevisiae*	Nucleus

protein 18			
25S rRNA (adenine(645)-N(1))-Methyltransferase	P38961	*Saccharomyces cerevisiae*	Nucleus
DNA replication ATP-dependent helicase/nuclease DNA2	P38859	*Saccharomyces cerevisiae*	Nucleus
Ribonuclease P protein subunit RPR2	P40571	*Saccharomyces cerevisiae*	Nucleus
DNA-directed RNA polymerase subunit	A0A8J5TW36	*Saccharomyces cerevisiae*	Nucleus
Zn(2)-C6 fungal-type domain-containing protein	X0CN47	*Fusarium oxysporum*	Nucleus
Fungal_trans domain-containing protein	W9JP90	*Fusarium oxysporum*	Nucleus
40S ribosomal protein S1	W9J4B8	*Fusarium oxysporum*	Cytoplasm
RNA helicase	A0A420QQN4	*Fusarium oxysporum*	Ribonucleoprotein complex
54S ribosomal protein L31, mitochondrial	A0A6M7ZGN8	*Fusarium oxysporum*	Mitochondrion
Small nuclear ribonucleoprotein Sm D2	W9J1T5	*Fusarium oxysporum*	Nucleus
U3 small nucleolar RNA-associated protein 10	W9KN02	*Fusarium oxysporum*	Nucleus
DNA polymerase gamma	P15801	*Saccharomyces cerevisiae*	Mitochondrion
54S ribosomal protein YmL6, Mitochondrial	P51998	*Saccharomyces cerevisiae*	Mitochondrion

GTP:AMP phosphotransferase, Mitochondrial	P26364	*Saccharomyces cerevisiae*	Mitochondrion matrix
ATPase synthesis protein 25, mitochondrial	A1DIN7	*Neosartorya fischeri*	Mitochondrion inner membrane
RNA exonuclease 3	Q4WYA1	*Neosartorya fumigata*	Nucleus
Cytoplasmic tRNA 2-thiolation protein 1	Q5AML2	*Candida albicans*	Cytoplasm
DNA (cytosine-5-)-methyltransferase DMT5	G2WR64	*Verticillium dahliae*	Nucleus
Transcription elongation factor SPT5	P0CR71	*Cryptococcus neoformans*	Nucleus
ATP-dependent RNA helicase DBP7	P0CQ95	*Cryptococcus neoformans*	Nucleus
Protein pyrABCN	Q08548	*Emericella nidulans*	Cytoplasm
ATPase synthesis protein 25, mitochondrial	Q09726	*Schizosaccharomyces pombe*	Mitochondrion inner membrane
Crossover junction endonuclease MUS81	Q04149	*Saccharomyces cerevisiae*	Nucleus
Nonsense-mediated mRNA decay protein 2	P38798	*Saccharomyces cerevisiae*	Cytoplasm
Endoribonuclease YSH1	Q4PEJ3	*Ustilago maydis*	Nucleus
mRNA-capping enzyme subunit alpha	Q01159	*Saccharomyces cerevisiae*	Nucleus
Protein BFR2	Q6FSD4	*Candida glabrata*	Nucleus

Lipid Metabolism

Lysophospholipid acyltransferase	Q08548	*Saccharomyces cerevisiae*	Endoplasmic reticulum membrane
Oxysterol-binding protein homolog 3	P38713	*Saccharomyces cerevisiae*	Cytoplasm
1-phosphatidylinositol 4,5-bisphosphate phosphodiesterase 1	P32383	*Fusarium oxysporum*	Chromosome, centromeric region
Inositol-3-phosphate synthase	P11986	*Saccharomyces cerevisiae*	Cytoplasm
Phosphatidyl-N-methylethanolamine N-methyltransferase	P05375	*Saccharomyces cerevisiae*	Endoplasmic reticulum membrane
Sphingosine-1-phosphate lyase	Q05567	*Saccharomyces cerevisiae*	Endoplasmic reticulum membrane
Cardiolipin synthase (CMP-forming)	Q07560	*Saccharomyces cerevisiae*	Mitochondrion inner membrane
Carrier domain-containing protein	A0A420REA3	*Saccharomyces cerevisiae*	Endoplasmic reticulum
NADPH--cytochrome P450 reductase	A0A3L6P0D2	*Fusarium oxysporum*	Endoplasmic reticulum
GP-PDE domain-containing protein	A0A8H4ZPR7	*Fusarium oxysporum*	Nucleus
Phosphatidylethanolamine N-methyltransferase	C5GN10	*Ajellomyces dermatitidis*	Endoplasmic reticulum membrane
O-acyltransferase ausQ	A0A1U8QLK0	*Emericella nidulans*	Nucleus
Lipoyl synthase, mitochondrial	A3GGJ5	*Scheffersomyces stipitis*	Mitochondrion
Lipase 5	Q9P8W0	*Candida albicans*	Secreted
Lovastatin diketide	Q9Y7D5	*Aspergillus terreus*	Unknown

synthase lovF			
Increased rDNA silencing Protein 4	Q6FJW0	*Candida glabrata*	Nucleus
3-hydroxy-3-methylglutaryl-coenzyme A reductase	B2KX91	*Ganoderma lucidum*	Nucleus
Acyl-protein thioesterase 1	Q12354	*Saccharomyces cerevisiae*	Cytoplasm
Hydroxymethylglutaryl-CoA synthase	K7PL94	*Ganoderma lucidum*	Cytoplasm
Lipoyl synthase, mitochondrial	P0CH68	*Candida albicans*	Mitochondrion
FAD-linked oxidoreductase dpmaF	P9WEY2	*Metarhizium anisopliae*	Cytoplasm
Dihydroxyacetone kinase	O74192	*Komagataella pastoris*	Cytoplasm
Acetyl-CoA carboxylase dmxL1	A0A4P8DJE6	*Cryptosporiopsis sp.*	Nucleus
Acetyl-coenzyme A synthetase	P16929	*Neurospora crassa*	Cytoplasm
Acyl-coenzyme A oxidase	Q756A9	*Ashbya gossypii*	Peroxisome
Carbohydrate metabolism			
Mannitol dehydrogenase 2	P0CX09	*Saccharomyces cerevisiae*	Cytoplasm
Alpha-L-rhamnosidase	A0A2H3TSB5	*Fusarium oxysporum*	Cytoplasm
GH16 domain-containing protein	A0A8H5A268	*Fusarium oxysporum*	Mitochondrion
alpha-1,2-Mannosidase	A0A6M7YP62	*Fusarium oxysporum*	Membrane

Glycerol-3-phosphate dehydrogenase [NAD(+)]	W9IWZ5	*Fusarium oxysporum*	glycerol-3-phosphate dehydrogenase complex
Glyco_transf_28 domain-containing protein	A0A8H6LSD4	*Fusarium oxysporum*	Cytoplasm
UDP-glucose 4-epimerase	W9JR32	*Fusarium oxysporum*	Cytoplasm
Alpha-L-arabinofuranosidase	B6F261	*Fusarium oxysporum*	Secreted
Cellulase domain-containing protein	W9I5E6	*Fusarium oxysporum*	Membrane
Glycerol-3-phosphate dehydrogenase [NAD(+)] 2, mitochondrial	P41911	*Saccharomyces cerevisiae*	Cytoplasm
Endochitinase 3 (Fragment)	Q6QDR4	*Metarhizium anisopliae*	Secreted
Lactam utilization protein lamB	P38096	*Emericella nidulans*	Cytoplasm
Regulation Factor or Signaling			
Serine/threonine-protein kinase PTK2/STK2	P47116	*Saccharomyces cerevisiae*	Nucleus
Regulator of Ty1 transposition protein 103	Q05543	*Saccharomyces cerevisiae*	Nucleus
Radiation-sensitive protein 28	Q12021	*Saccharomyces cerevisiae*	Nucleus
GATA-type domain-containing protein	W9KH05	*Fusarium oxysporum*	Nucleus
Ino eighty subunit 2	P40154	*Saccharomyces cerevisiae*	Nucleus
Clustered mitochondria protein 1	Q03690	*Saccharomyces cerevisiae*	Cytoplasm
DNA cross-link repair protein PSO2/SNM1	P30620	*Saccharomyces cerevisiae*	Nucleus
Protein SST2	P11972	*Saccharomyces cerevisiae*	Membrane

Aminodeoxychorismate synthase	P37254	*Saccharomyces cerevisiae*	Cytoplasm
Transcription initiation factor TFIID subunit 1	P46677	*Saccharomyces cerevisiae*	Nucleus
NuA3 HAT complex component NTO1	Q12311	*Saccharomyces cerevisiae*	Nucleus
Protein phosphatase PP2A regulatory subunit A	P31383	*Saccharomyces cerevisiae*	Cytoplasm/ Nucleus
Tyrosyl-DNA phosphodiesterase 1	P38319	*Saccharomyces cerevisiae*	Cytoplasm/ Nucleus
Silencing boundary-establishment protein FUB1	P25659	*Saccharomyces cerevisiae*	Nucleus
E3 ubiquitin-protein ligase complex SLX5-SLX8 subunit SLX5	P32828	*Saccharomyces cerevisiae*	Nucleus
Respiration factor 2	P46974	*Saccharomyces cerevisiae*	Nucleus
APC/C-CDH1 modulator 1	Q08981	*Saccharomyces cerevisiae*	Nucleus
Ribosome-interacting GTPase 1	P53295	*Saccharomyces cerevisiae*	Cytoplasm
Casein kinase II subunit beta'	P43639	*Saccharomyces cerevisiae*	Cytoplasm/Nucleus
Type 1 phosphatases regulator YPI1	P43587	*Saccharomyces cerevisiae*	Nucleus
DNA repair protein XRS2	P33301	*Saccharomyces cerevisiae*	Nucleus
Regulatory protein SWI4	P25302	*Saccharomyces cerevisiae*	Nucleus

SWIRM domain-containing protein FUN19	P28003	*Saccharomyces cerevisiae*	Nucleus
Cytosolic Fe-S cluster assembly factor CFD1	P40558	*Saccharomyces cerevisiae*	Nucleus
F-box protein COS111	P38308	*Saccharomyces cerevisiae*	Mitochondrion
HDA1 complex subunit 2	Q06629	*Saccharomyces cerevisiae*	Nucleus
Serine/threonine-protein kinase Tel1	F9G3R4	*Saccharomyces cerevisiae*	Chromosome
1-phosphatidylinositol 4-kinase	W9LB23	*Fusarium oxysporum*	Nucleus/Cytoplasm
RFX-type winged-helix domain-containing protein	W9KMD6	*Fusarium oxysporum*	Nucleus
Histone deacetylase	A0A0D2YC83	*Fusarium oxysporum*	Nucleus
INCENP_ARK-bind domain-containing protein	W9KH71	*Fusarium oxysporum*	Nucleus
Phosphatidylinositol 3-kinase VPS34	A0A0D2XHC6	*Fusarium oxysporum*	Nucleus
Polyadenylate-binding protein	W9I1Q9	*Fusarium oxysporum*	Cytoplasm
BZIP domain-containing protein	A0A6M7Z7J4	*Fusarium oxysporum*	Nucleus
26S proteasome regulatory subunit RPN1	W9KBT4	*Fusarium oxysporum*	Proteosoma
Mediator of RNA polymerase II transcription subunit 5	A0A0D2XC79	*Fusarium oxysporum*	Nucleus

Histone transcription regulator 3 homolog	Q4IFH8	*Gibberella zeae*	Nucleus
Mediator of RNA polymerase II transcription subunit 7	Q5AEN6	*Candida albicans*	Nucleus
Guanine nucleotide-binding protein subunit	O74227	*Cochliobolus heterostrophus*	heterotrimeric G-protein complex
Multiprotein-bridging factor 1	Q52BY4	*Magnaporthe oryzae*	Nucleus
CCR4-Not complex 3'-5'-exoribonuclease subunit Ccr4	Q9C2R2	*Neurospora crassa*	Nucleus
Transcriptional regulatory protein PHO23	P50947	*Saccharomyces cerevisiae*	Nucleus
Protein kinase C-like	P87253	*Neurospora crassa*	Cell septum
Signal recognition particle receptor subunit beta	P36057	*Saccharomyces cerevisiae*	Endoplasmic reticulum membrane
Cutinase transcription factor 1 alpha	P52958	*Fusarium vanettenii*	Nucleus
TATA-binding protein-associated factor mot1	P32333	*Saccharomyces cerevisiae*	Nucleus
EKC/KEOPS complex subunit BUD32	B7XIB8	*Enterocytozoon bieneusi*	*Chromosome*
GPI ethanolamine phosphate transferase 1	Q6CW36	*Kluyveromyces lactis*	Endoplasmic reticulum membrane
Transcription regulation			
Transcription factor CSR2	Q12734	*Saccharomyces cerevisiae*	Nucleus

MFS domain-containing protein	W9KH71	*Fusarium oxysporum*	Cytoplasm
Mediator of RNA polymerase II transcription subunit 5	Q4PAB8	*Ustilago maydis*	Nucleus
Cell division			
Anaphase-promoting complex subunit CDC16	P09798	*Saccharomyces cerevisiae*	Nucleus
Amino acid metabolism			
Glycine dehydrogenase (decarboxylating), mitochondrial	P26969	*Saccharomyces cerevisiae*	Mitochondrion
Pyruvate decarboxylase isozyme 3	P11986	*Saccharomyces cerevisiae*	Cytoplasm
Cystathionine beta-synthase	P32582	*Saccharomyces cerevisiae*	Cytoplasm
Acetolactate synthase small subunit, mitochondrial	P25605	*Saccharomyces cerevisiae*	Mitochondrion
Pyruvate decarboxylase isozyme 2	P16467	*Saccharomyces cerevisiae*	Cytoplasm
Alanine--tRNA ligase	W9L4A1	*Fusarium oxysporum*	Mitochondrion
Imidazoleglycerol-phosphate dehydratase	W9J9C7	*Fusarium oxysporum*	Mitochondrion
D-3-phosphoglycerate dehydrogenase	W9KMP9	*Fusarium oxysporum*	Cytoplasm
C-1-tetrahydrofolate synthase, mitochondrial	O43007	*Saccharomyces pombe*	Mitochondrion

Arginine biosynthesis bifunctional protein ArgJ, mitochondrial	Q6C627	*Yarrowia lipolytica*	Mitochondrion
Methylthioribulose-1-phosphate dehydratase	B6QQ13	*Talaromyces marneffei*	Cytoplasm
Pentafunctional AROM polypeptide	B2B223	*Podospora anserina*	Cytoplasm
Copper transport protein	P25355	*Saccharomyces cerevisiae*	Cytoplasm
3-isopropylmalate dehydratase	Q74ZM9	*Ashbya gossypii*	Cytoplasm
Histidine biosynthesis trifunctional protein	O74712	*Candida albicans*	Cytoplasm
Arginine biosynthesis bifunctional protein ArgJ, mitochondrial	A0A5N6H9B7	*Aspergillus flavus*	Cytoplasm
Stress and Defense Response			
mitochondrial hydrolase	P53889	*Saccharomyces cerevisiae*	Mitochondrion
oxidoreductase C24B10.20	Q9P7I6	*Schizosaccharomyces pombe*	Cytoplasm
Glutathione peroxidase-like peroxiredoxin gpx1	O59858	*Schizosaccharomyces pombe*	Cytoplasm
Pathogenicity			
Acetyltransferase sirH	Q6Q889	*Leptosphaeria maculans*	Membrane
Mitogen-activated protein kinase kinae kinase MST11	G4N7X0	*Magnaporthe oryzae*	Nucleus

Subtilisin-like protease 6	A1XIH0	*Trichophyton equinum*	Secreted
Other Metabolites			
Thiamine biosynthetic bifunctional enzyme	P41835	*Saccharomyces cerevisiae*	Cytoplasm
FAD_binding_3 domain-containing protein	A0A6M7Z3Q6	*Fusarium oxysporum*	Membrane
Amine oxidase	F9FXV0	*Fusarium oxysporum*	Cytoplasm
Tyrosinase	A0A6M7ZIT1	*Fusarium oxysporum*	Endoplasmic reticulum /Golgi
Iterative polyketide synthase afoE	Q5BEJ6	*Emericella nidulans*	Endoplasmic reticulum
Molybdenum cofactor sulfurase	B0Y691	*Neosartorya fumigata*	Cytoplasm
oxidoreductase C30D10.05c	O14351	*Saccharomyces pombe*	Cytoplasm
Adenylyltransferase and sulfurtransferase uba4	A4RPM5	*Magnaporthe oryzae*	Cytoplasm
Hybrid PKS-NRPS synthetase iliA	P0DO30	*Neonectria sp.*	Endoplasmic reticulum membrane
Halogenase otaD	A2R6G7	*Aspergillus niger*	Mitochondrion
Dehydrogenase xptC	Q5AUN2	*Emericella nidulans*	Mitochondrion
Cytochrome P450 monooxygenase atnE	Q5AUZ9	*Emericella nidulans*	Membrane
Unknown			
NADP-dependent 3-hydroxy	Q05016	*Saccharomyces cerevisiae*	Cytoplasm

acid dehydrogenase			
UPF0641 membrane protein YHR140W	P38842	*Saccharomyces cerevisiae*	Endoplasmic reticulum membrane
Protein FYV8	P46949	*Saccharomyces cerevisiae*	Endoplasmic reticulum membrane
Protein SGM1	Q06629	*Saccharomyces cerevisiae*	Nucleus
FAD-binding PCMH-type domain-containing protein	A0A6M7YWV0	*Fusarium oxysporum*	Nucleus
HpcH_HpaI domain-containing protein	A0A6M7Z6R5	*Fusarium oxysporum*	Mitochondrion
Transket_pyr domain-containing protein	A0A6M7Z6R5	*Fusarium oxysporum*	Mitochondrion
NB-ARC domain-containing protein	A0A420MRP4	*Fusarium oxysporum*	Cytoplasm
COesterase domain-containing protein	X0H0X2	*Fusarium oxysporum*	Nucleus
RNB domain-containing protein	A0A0D2XD23	*Fusarium oxysporum*	Nucleus
AB hydrolase-1 domain-containing protein	X0HJI4	*Fusarium oxysporum*	Cytoplasm
OTU domain-containing protein	W9JXF0	*Fusarium oxysporum*	Nucleus
C2H2-type domain-containing protein	W9JIH0	*Fusarium oxysporum*	Nucleus
Amidase	W9K8T3	*Fusarium oxysporum*	Cytoplasm

HEME_HALOPEROXIDASE domain-containing protein	A0A2H3U0R6	*Fusarium oxysporum*	Nucleus
Ribonuclease Z	A0A8H4Z2B5	*Fusarium oxysporum*	Cytoplasm
GMC_OxRdtase_N domain-containing protein	W9I4W9	*Fusarium oxysporum*	Cytoplasm
AB hydrolase-1 domain-containing protein	A0A6M7ZKL2	*Fusarium oxysporum*	Nucleus
HET domain-containing protein	X0B8E0	*Fusarium oxysporum*	Cytoplasm
Hydrolase_4 domain-containing protein	A0A2H3SLL2	*Fusarium oxysporum*	Cytoplasm
Amidohydro-rel domain-containing protein	A0A2H3SLL2	*Fusarium oxysporum*	Peroxisomal
Fido domain-containing protein	A0A420RA84	*Fusarium oxysporum*	Nucleus
Aldedh domain-containing protein	F9F287	*Fusarium oxysporum*	Cytoplasm
NACHT domain-containing protein	A0A6M7Z9K2	*Fusarium oxysporum*	Nucleus
NAD(+) diphosphatase	A0A559KVW1	*Fusarium oxysporum*	Nucleus
P4Hc domain-containing Protein	A0A0D2XZJ7	*Fusarium oxysporum*	Cytoplasm
Methyltransf_25 domain-containing protein	W9JMD3	*Fusarium oxysporum*	Mitochondrion

Translation machinery-associated protein 22	A2QHG9	*Aspergillus niger*	Cytoplasm
Siderophore transporter MYCGRDRAFT_70577	F9X9V3	*Zymoseptoria tritici*	Membrane
Malformin synthetase mlfA	A0A1L9NGU5	*Aspergillus tubingensis*	Endoplasmic reticulum membrane

Supplementary Table S2 – Unique proteins identified in the treated group by ESI-LC-MS/MS

Protein Name	ID (Uniprot)	Organism Reference	Cellular Compartment
Transport			
Vacuolar protein-sorting-associated protein 46	P69771	*Saccharomyces cerevisiae*	Endossome membrane
Autophagy-related protein 9	Q06628	*Saccharomyces cerevisiae*	Membrane
Mitochondrial import inner membrane translocase subunit TIM23	P32897	*Saccharomyces cerevisiae*	Mitochondrion inner membrane
Low affinity vacuolar monovalent cation/H (+) antiporter	P42839	*Saccharomyces cerevisiae*	Vacuole membrane
GTP-binding protein GTR1	Q00582	*Saccharomyces cerevisiae*	Vacuole membrane
Protein SNQ2	P32568	*Saccharomyces cerevisiae*	Membrane
Intracellular protein transport protein	P25386	*Saccharomyces cerevisiae*	Endoplasmic reticulum/Golgi apparatus
Vacuolar protein sorting/targeting protein VPS	P54787	*Saccharomyces cerevisiae*	Golgi apparatus
Hexose transporter HXT9	P40885	*Saccharomyces cerevisiae*	Membrane
Conserved oligomeric Golgi complex subunit 6	P54787	*Saccharomyces cerevisiae*	Golgi membrane
Membrane-anchored lipid-binding protein LAM6	Q08001	*Saccharomyces cerevisiae*	Endoplasmic reticulum membrane
GTP-binding protein YPT32/YPT11	P51996	*Saccharomyces cerevisiae*	Golgi apparatus membrane

MFS domain-containing protein	A0A5C6SDT0	*Fusarium oxysporum*	Membrane
Importin N-terminal domain-containing Protein	A0A0D2XQA6	*Fusarium oxysporum*	Nucleus
FAD-binding FR-type domain-containing Protein	W9HR83	*Fusarium oxysporum*	Membrane
GPI ethanolamine phosphate transferase	A0A420Q1C6	*Fusarium oxysporum*	Endoplasmic reticulum
Bifunctional cytochrome P450/NADPH--P450 reductase	Q9Y8G7	*Fusarium oxysporum*	Nucleus / cytoplasm
MFS domain-containing protein	A0A5C6SDT0	*Fusarium oxysporum*	Membrane
Adenylyltransferase AND sulfurtransferase uba4	A0A0D2XXF9	*Fusarium oxysporum*	Cytosol
MFS domain-containing protein	A0A5C6SDT0	*Fusarium oxysporum*	Membrane
Cation_ATPase_N domain-containing protein	A0A8H5ALP8	*Fusarium oxysporum*	Membrane
Proton-translocating NAD(P)(+) transhydrogenase	P73496	*Fusarium oxysporum*	Membrane
Serine/threonine-protein kinase ATG1	Q52EB3	*Magnaporthe oryzae*	Cytoplasm
DnaJ-related protein spj1	O94625	*Schizosaccharomyces pombe*	Endoplasmic reticulum
Mating factor M secretion protein mam1	P78966	*Schizosaccharomyces pombe*	Membrane
Protein SMY2	P32909	*Saccharomyces cerevisiae*	Cytoplasm

Low-affinity phosphate transporter PHO91	P27514	*Saccharomyces cerevisiae*	Vacuole membrane
Peroxisomal membrane protein PAS20	P80667	*Saccharomyces cerevisiae*	Peroxisome membrane
CRAL-TRIO domain-containing protein C23B6.04c	Q9UU99	*Schizosaccharomyces pombe*	Cell membrane
Copper transport protein ctr4	O94722	*Schizosaccharomyces pombe*	Membrane
Plasma membrane ATPase 1	P09627	*Schizosaccharomyces pombe*	Cell membrane
Presequence translocated-associated motor subunit pam17, mitochondrial	C8VTR5	*Emericella nidulans*	Mitochondrion inner membrane
Vacuolar protein-sorting protein bro-1	Q7SAN9	*Neurospora crassa*	Endosome
ATPase GET3	Q6CPX3	*Kluyveromyces lactis*	Cytoplasm
Vacuolar protein sorting/targeting protein 10	C5FYX2	*Arthroderma otae*	Golgi apparatus
Major glycerophosphoinositol permease GIT3	Q5A1L6	*Candida albicans*	Cell membrane
Plasma membrane ATPase	P24545	*Zygosaccharomyces rouxii*	Cell membrane

Cell Function and Structure

Mitotic check point protein BFA1	P47113	*Saccharomyces cerevisiae*	Cytoplasm
ATP-dependent DNA helicase CHL1	P22516	*Saccharomyces cerevisiae*	Nucleus

Actin-related protein 2	P32381	*Saccharomyces cerevisiae*	Cytoplasm
Growth regulation protein	P12611	*Saccharomyces cerevisiae*	Nucleus
Serine/threonine-protein kinase YPK2/YKR2	P18961	*Saccharomyces cerevisiae*	Cytoplasm
Protein UIP5	P36137	*Saccharomyces cerevisiae*	Nucleus membrane
Nucleoporin NUP159	P40477	*Saccharomyces cerevisiae*	Nucleus
Protein TMA23	Q03525	*Saccharomyces cerevisiae*	Nucleus
Exocyst complex component SEC8	O74562	*Saccharomyces cerevisiae*	Cytoplasm
Protein STU1	P38198	*Saccharomyces cerevisiae*	Centromere
Protein PAL1	Q05518	*Saccharomyces cerevisiae*	Membrane
Serine/threonine-protein kinase CLA4	P48562	*Saccharomyces cerevisiae*	Cytoplasm, cytoskeleton
Sister chromatid cohesion protein 1	P40090	*Saccharomyces cerevisiae*	Nucleus
Essential for maintenance of the cell wall protein 1	P42842	*Saccharomyces cerevisiae*	Cytoplasm
Serine/threonine-protein kinase GIN4	Q12263	*Saccharomyces cerevisiae*	Cytoplasm
Phosphatidylinositol 4,5-bisphosphate 5-phosphatase INP51	P40559	*Saccharomyces cerevisiae*	Cytoplasm
Monopolar spindle protein 2	P53159	*Saccharomyces cerevisiae*	Cytoplasm
Chitin synthase	Q873Z8	*Fusarium oxysporum*	Cytoplasm

Aldedh domain-containing protein	F9F287	*Fusarium oxysporum*	Cytoplasm
Protein SPA2	P23201	*Saccharomyces cerevisiae*	Cell tip
ASTRA-associated protein 1	A7ESR0	*Sclerotinia sclerotiorum*	Nucleus
Pheromone-processing carboxypeptidase kex1	B6K7U7	*Schizosaccharomyces japonicus*	Golgi apparatus / trans-Golgi network membrane
Autophagy-related protein 2	A2QSC9	*Aspergillus niger*	Endoplasmic reticulum membrane
Inner kinetochore subunit CTF19	Q02732	*Saccharomyces cerevisiae*	Nucleus
Autophagy-related protein 9	Q75A48	*Saccharomyces cerevisiae*	Preautophagosomal structure membrane
Serine/threonine- protein kinase tor2	Q9Y7K2	*Schizosaccharomyces pombe*	Cytoplasm
Anaphase-promoting complex subunit 2	Q12440	*Saccharomyces cerevisiae*	Cytoplasm
GPI ethanolamine phosphate transferase 1	Q4ILH3	*Gibberella zeae*	Endoplasmic reticulum membrane
Aspartic proteinase yapsin-7	Q06325	*Saccharomyces cerevisiae*	Cytoplasm
Inactive metallocarboxypeptidase ecm14	B8M2K0	*Talaromyces stipitatus*	Vacuole
MICOS complex subunit mic60	Q7SFD8	*Neurospora crassa*	Mitochondrion inner membrane
Sporulation-specific protein 22	P40511	*Saccharomyces cerevisiae*	Cell membrane
Energetic metabolism			
Succinate dehydrogenase assembly factor 3, mitochondrial	Q04401	*Saccharomyces cerevisiae*	Mitochondrion

Cytochrome c mitochondrial import factor CYC2	P38909	*Saccharomyces cerevisiae*	Mitochondrion inner membrane
Alcohol dehydrogenase 4	P10127	*Saccharomyces cerevisiae*	Mitochondrion
54S ribosomal protein L17, mitochondrial	P36528	*Saccharomyces cerevisiae*	Mitochondrion
NAD-dependent malic enzyme, mitochondrial	P36013	*Saccharomyces cerevisiae*	Mitochondrion
Glycogen debrancher	A0A2H3T8G3	*Fusarium oxysporum*	Cytoplasm
Kinesin motor domain-containing protein	W9L7Z1	*Fusarium oxysporum*	Mitochondrion
Structural maintenance of chromosomes Protein	A0A0D2XQL5	*Fusarium oxysporum*	Nucleus
Kinesin-like protein	A0A6M7Z0I0	*Fusarium oxysporum*	Microtubule
HTH APSES-type domain-containing Protein	W9KJX9	*Fusarium oxysporum*	Nucleus
Glycerol kinase	W9JLB5	*Fusarium oxysporum*	Cytoplasm
Isoamyl acetate-hydrolyzing esterase	O74648	*Schizosaccharomyces pombe*	Cytoplasm
ATP synthase subunit 5, mitochondrial	A0A6A5Q1Y3	*Saccharomyces cerevisiae*	Mitochondrion inner membrane
Phosphoglycerate kinase 2	P29406	*Rhizopus niveus*	Cytoplasm
NADH-ubiquinone oxidoreductase chain 4	A9RAH8	*Debaryomyces hansenii*	Mitochondrion membrane

Protein Folding and metabolism

E3 ubiquitin-protein ligase substrate receptor	P39940	*Saccharomyces cerevisiae*	Cytoplasm
Isoleucine--tRNA ligase, cytoplasmic	P09436	*Saccharomyces cerevisiae*	Cytoplasm
N-terminal acetyltransferase A complex subunit NAT1	P12945	*Saccharomyces cerevisiae*	Cytoplasm
N-alpha-acetyltransferase 35, NatC auxiliary subunit	Q02197	*Saccharomyces cerevisiae*	Cytoplasm
5-demethoxyubiquinone hydroxylase, mitochondrial	P41735	*Saccharomyces cerevisiae*	Mitochondrion inner membrane
CUE domain-containing protein CUE4	Q04201	*Saccharomyces cerevisiae*	Cytoplasm
Low molecular weight phosphotyrosine protein phosphatase	P40347	*Saccharomyces cerevisiae*	Cytoplasm
H/ACA ribonucleoprotein complex non-core subunit NAF1	P53919	*Saccharomyces cerevisiae*	Nucleus
Elongation factor 3B	P53978	*Saccharomyces cerevisiae*	Cytoplasm
Coupling of ubiquitin conjugation to ER degradation protein 1	Q08412	*Saccharomyces cerevisiae*	Endoplasmic reticulum membrane

Actin cytoskeleton-regulatory complex protein PAN1	P32521	*Saccharomyces cerevisiae*	Endosome membrane
Ribosome biogenesis protein UTP30	P36144	*Saccharomyces cerevisiae*	Nucleus
Dolichyldiphosphatase	P53223	*Saccharomyces cerevisiae*	Endoplasmic reticulum membrane
Proteasome chaperone 1	P53196	*Saccharomyces cerevisiae*	Cytoplasm
Dipeptidase	A0A2H3T5I7	*Fusarium oxysporum*	Cytoplasm
Peptidase_S8 domain-containing protein	A0A420NBM7	*Fusarium oxysporum*	Nucleus
Leukotriene A-4 hydrolase homolog	W9ZSS5	*Fusarium oxysporum*	Endoplasmic reticulum
Protein kinase domain-containing Protein	A0A0D2XQE2	*Fusarium oxysporum*	Nucleus
Protein kinase domain-containing protein	A0A0D2XQE2	*Fusarium oxysporum*	Cytoplasm
Protein kinase domain-containing protein	A0A0D2XQE2	*Fusarium oxysporum*	Nucleus
54S ribosomal protein yml6, mitochondrial	O74801	*Schizosaccharomyces pombe*	Mitochondrion
Methionine aminopeptidase 2	E3L3Q8	*Puccinia graminis*	Cytoplasm
Ubiquinone biosynthesis protein COQ4, mitochondrial	A5DVZ3	*Lodderomyces elongisporus*	Mitochondrion inner membrane
DUB-associated factor 1	Q99247	*Saccharomyces cerevisiae*	Cytoplasm
Isoleucine--tRNA ligase, mitochondrial	Q9USL3	*Schizosaccharomyces pombe*	Mitochondrion matrix
Lon protease homolog 2, peroxisomal	Q9USL3	*Neurospora crassa*	Peroxisomal matrix

Protein OS-9 homolog	Q756T2	*Ashbya gossypii*	Endoplasmic reticulum membrane
Eukaryotic translation initiation factor 6	A0A0B0DUG9	*Neurospora crassa*	Nucleus / nucleolus
Alanine--tRNA ligase	Q5AQL1	*Emericella nidulans*	Mitochondrion
Osmolarity two-component system protein SSK1	Q07084	*Saccharomyces cerevisiae*	Cytoplasm
Dol-P-Man:Man(5)GlcNAc(2)-PP-Dol alpha-1,3-mannosyltransferase	0TVF9	*Phaeosphaeria nodorum*	Endoplasmic reticulum membrane
Eukaryotic translation initiation factor 3 subunit I	P79083	*Schizosaccharomyces pombe*	Cytoplasm
U3 small nucleolar RNA-associated protein 12	Q12220	*Saccharomyces cerevisiae*	Nucleus / nucleolus
Zuotin	Q9Y7I8	*Schizosaccharomyces pombe*	Cytosol

DNA and RNA binding

ATP-dependent RNA helicase ROK1	P45818	*Saccharomyces cerevisiae*	Nucleus
Protein URA2	P07259	*Saccharomyces cerevisiae*	Cytoplasm
RNA exonuclease 1	Q12149	*Saccharomyces cerevisiae*	Nucleus
Pre-rRNA-processing protein IPI1	P38803	*Saccharomyces cerevisiae*	Nucleus
Protein MPE1	P35728	*Saccharomyces cerevisiae*	Nucleus

Cytoplasmic 60S subunit biogenesis factor REI1	P38344	*Saccharomyces cerevisiae*	Cytoplasm
U4/U6 snRNA-associated-splicing factor PRP24	P49960	*Saccharomyces cerevisiae*	Nucleus
5'-deoxynucleotidase YBR242W	P38331	*Saccharomyces cerevisiae*	Cytoplasm
tRNA (cytidine(32)-2'-O)-methyltransferase non-catalytic subunit TRM732	P38238	*Saccharomyces cerevisiae*	Cytoplasm
Mitochondrial group I intron splicing factor CCM1	P48237	*Saccharomyces cerevisiae*	Mitochondrion
H/ACA ribonucleoprotein complex subunit CBF5	P33322	*Saccharomyces cerevisiae*	Nucleus
DNA-directed RNA polymerase subunit	A0A0C4DHV9	*Fusarium oxysporum*	Nucleus
Zn(2)-C6 fungal-type domain-containing Protein	A0A0D2XDM8	*Fusarium oxysporum*	Nucleus
DNA topoisomerase I	A0A2C8CY01	*Fusarium oxysporum*	Chromosome
Kynurenine 3-monooxygenase	A0A2H3SUS1	*Fusarium oxysporum*	Mitochondrion outer membrane
Zn(2)-C6 fungal-type domain-containing protein	A0A0D2XDM8	*Fusarium oxysporum*	Nucleus
DNA-directed RNA polymerase subunit beta	A0A0C4DHV9	*Fusarium oxysporum*	Nucleus
DNA topoisomerase 2	A0A6M7YWD6	*Fusarium oxysporum*	Nucleus

Fungal_trans domain-containing Protein	W9JP90	*Fusarium oxysporum*	Nucleus
40S ribosomal protein S26	W9I0R7	*Fusarium oxysporum*	Ribosome
CTP synthase	A0A6M7YU47	*Fusarium oxysporum*	Mitochondrion /cytoplasm
eIF-2B GDP-GTP exchange factor subunit epsilon	X0LIC3	*Fusarium oxysporum*	Nucleus
Orotate phosphoribosyltransferase	Q1DNB0	*Coccidioides immitis*	Mitochondrion
Flap endonuclease 1	A5E121	*Lodderomyces elongisporus*	Nucleus/nucleolus
mRNA-capping enzyme subunit alpha	Q01159	*Schizosaccharomyces pombe*	Nucleus
Histone H4	Q6ZXX3	*Ustilago maydis*	Nucleosome
60S ribosomal protein	O13418	*L15 Aspergillus niger*	Ribosome
tRNA (uracil-O(2)-)-methyltransferase	Q6BRY1	*Debaryomyces hansenii*	Cytoplasm
U3 small nucleolar RNA-associated protein 10	A5E212	*Lodderomyces elongisporus*	Nucleus/nucleolus/membrane
60S ribosomal protein L7	Q6FSN6	*Candida glabrata*	Cytosolic large ribosomal subunit
Zinc finger CCCH domain-containing protein C337.12	O74823	*Schizosaccharomyces pombe*	Nucleus
Polyadenylate-binding protein, cytoplasmic and nuclear	A5DM21	*Meyerozyma guilliermondii*	Nucleus/cytoplasm
ATP-dependent RNA helicase MRH4, mitochondrial	Q755C5	*Ashbya gossypii*	Mitochondrion
tRNA (adenine(58)-N(1))-methyltransferase non-catalytic subunit TRM6	Q6CF35	*Yarrowia lipolytica*	Nucleus
Nucleolar protein 58	A2QE38	*Aspergillus niger*	Nucleus/nucleolus

ATP-dependent RNA helicase ROK1	P45818	*Saccharomyces cerevisiae*	Nucleolus
Inosine triphosphate pyrophosphatase	D5GCI8	*Tuber melanosporum*	Cytoplasm
54S ribosomal protein L3, mitochondrial	O43042	*Schizosaccharomyces pombe*	Mitochondrion
tRNA (adenine(58)-N(1))-methyltransferase non-catalytic subunit trm6	Q4WE58	Neosartorya fumigata	Nucleus
Cytochrome b mRNA maturase bI3	A9RAG7	*Debaryomyces hansenii*	Mitochondrion inner membrane
Pre-mRNA-processing ATP-dependent RNA helicase PRP5	A5E058	*Lodderomyces elongisporus*	Nucleus
Dihydroorotate dehydrogenase (quinone), mitochondrial	Q63707	*Lachancea kluyveri*	Mitochondrion inner membrane
Mitochondrial escape protein 2	P32843	*Saccharomyces cerevisiae*	Mitochondrion inner membrane
Flap endonuclease 1	Q6BLF4	*Debaryomyces hansenii*	Nucleus/nucleolus
Orotidine 5'-phosphate decarboxylase	P15188	*Ustilago maydis*	Cytosol

<u>Lipid Metabolism</u>

Manganese lipoxygenase	F9FRH4	*Fusarium oxysporum*	Nucleus
PlsC domain-containing protein	A0A420SRZ6	*Fusarium oxysporum*	Cellular anatomical Entity

Carbohydrate metabolism

Phosphoenolpyruvate carboxykinase (ATP)	P10963	*Saccharomyces cerevisiae*	Cytoplasm
SNF1-activating kinase 1	P38990	*Saccharomyces cerevisiae*	Cytoplasm
Alpha-glucosidase MAL12	P53341	*Saccharomyces cerevisiae*	Membrane
Glucosamine-6-phosphate deaminase	W9KB79	*Fusarium oxysporum*	Cytoplasm
Cas1_AcylT domain-containing Protein	A0A6M7YXG0	*Fusarium oxysporum*	Membrane
Phosphoglycerate kinase	A0A1D8MJA9	*Fusarium oxysporum*	Nucleus / cytoplasm
Transcription activator of gluconeogenesis ERT1	C4YLC3	*Candida albicans*	Nucleus
Beta-mannosidase B	Q5B7W2	*Emericella nidulans*	Cytoplasm
NAD(P)H-dependent D-xylose reductase	Q9P430	*Scheffersomyces shehatae*	Cytoplasm
Exo-alpha-sialidase	Q4WQS0	*Neosartorya fumigata*	Cytoplasm
Ergosteryl-beta-glucosidase	P40566	*Saccharomyces cerevisiae*	Cytoplasm/cytosol
Cytosolic neutral trehalase	P52494	*Candida albicans*	Cytoplasm
FAD-binding monooxygenase prhJ	A0A1E1FFN7	*Penicillium brasilianum*	Cytoplasm / mitochondrion
Patatin-like phospholipase domain-containing protein MGG_12849	A4R8V2	*Magnaporthe oryzae*	Membrane

Lysophospholipase	Q2H0D3	*NTE1 Chaetomium globosum*	Endoplasmic reticulum
Non-reducing polyketide synthase stbA	A0A193PS74	*Stachybotrys bisbyi*	Nucleus
Triacylglycerol lipase ptl3	Q9Y827	*Schizosaccharomyces pombe*	Cytoplasm
Hybrid PKS-NRPS synthetase TAS1	G4N137	*Magnaporthe oryzae*	Mitochondrion
Very-long-chain 3-oxoacyl-CoA reductase	Q75A60	*Ashbya gossypii*	Endoplasmic reticulum membrane
NADPH--cytochrome P450 reductase	P16603	*Saccharomyces cerevisiae*	Endoplasmic reticulum membrane
Patatin-like phospholipase domain-containing protein CIMG_04897	Q1DXR6	*Coccidioides immitis*	Membrane
Fatty acid synthase subunit alpha	P43098	*Candida albicans*	Fatty acid synthase complex

Pathogenicity

Cyclochlorotine synthetase	A0A0U1LQE6	*Talaromyces islandicus*	Endoplasmic reticulum
Nonribosomal peptide synthetase 1	Q4WT66	*Neosartorya fumigata*	Cytoplasm
Nonribosomal peptide synthetase vlms	A0A024F910	*Lecanicillium*	Endoplasmic reticulum
Reducing polyketide synthase AFT16-1	V5Y0F7	*Alternaria alternata*	Nucleus / Cytoplasm
Acyltransferase BOA11	A6SSW1	*Botryotinia fuckeliana*	Cytoplasm

Regulation Factor or Signaling

Negative regulator of RAS-cAMP pathway	P34072	*Saccharomyces cerevisiae*	Nucleus

Protein SRL3	P36167	*Saccharomyces cerevisiae*	Cytoplasm
SWI/SNF global transcription activator complex subunit	P43554	*Saccharomyces cerevisiae*	Nucleus
Pheromone a factor receptor	Q00619	*Saccharomyces cerevisiae*	Membrane
MEMO1 family protein MHO1	P47085	*Saccharomyces cerevisiae*	Cytoplasm
General transcription and DNA repair factor IIH subunit TFB2	Q02939	*Saccharomyces cerevisiae*	Nucleus
Regulatory protein SWI6	P09959	*Saccharomyces cerevisiae*	Nucleus/Cytoplasm
Transcription regulatory protein SNF2	P22082	*Saccharomyces cerevisiae*	Nucleus
Rho-GTPase-activating protein RGD2	P43556	*Saccharomyces cerevisiae*	Nucleus
Protein PET122, mitochondrial	P10355	*Saccharomyces cerevisiae*	Mitochondrion inner membrane
Transcription factor TFIIIB component B"	P46678	*Saccharomyces cerevisiae*	Nucleus
Zinc finger protein MSN2	P33748	*Saccharomyces cerevisiae*	Cytoplasm/ Nucleus
DNA repair/transcription protein MET18/MMS19	P40469	*Saccharomyces cerevisiae*	Nucleus
Aprataxin-like protein	Q08702	*Saccharomyces cerevisiae*	Nucleus
Mitochondrial escape protein 2	P32843	*Saccharomyces cerevisiae*	Mitochondrion inner membrane
Actin-related protein 8	W9IT40	*Fusarium oxysporum*	Nucleus
Mediator of RNA polymerase II transcription subunit 1	A0A0D2XHG4	*Fusarium oxysporum*	Nucleus

Zn(2)-C6 fungal-type domain-containing Protein	A0A0D2XDM8	*Fusarium oxysporum*	Nucleus
ATP-dependent DNA helicase	A0A8H6LMY2	*Fusarium oxysporum*	Nucleus
Fork-head domain-containing protein	W9JSR5	*Fusarium oxysporum*	Nucleus
Serine/threonine-protein kinase TEL1	Q6CP76	*Kluyveromyces lactis*	Chromosome
Vacuolar membrane-associated protein iml1	Q2UMR9	*Aspergillus oryzae*	Vacuolar membrane
ISWI chromatin-remodeling complex ATPase ISW2	Q5A310	*Candida albicans*	Nucleus
DNA mismatch repair protein MSH3	Q6CHE5	*Yarrowia lipolytica*	Nucleus
C2H2 type master regulator of conidiophore development brlA	P10069	*Aspergillus oryzae*	Nucleus
Methylated-DNA--protein-cysteine methyltransferase	Q6BVY4	*Debaryomyces hansenii*	Nucleus
Verprolin	P37370	*Saccharomyces cerevisiae*	Cytoplasm/cytoskeleto
Nicotinate catabolism cluster-specific transcription factor	C8VJW0	*Emericella nidulans*	Nucleus
Transcription activator of gluconeogenesis HCBG_00867	C0NCM1	*Ajellomyces capsulatus*	Nucleus
Negative cofactor 2 complex subunit beta	Q92317	*Saccharomyces cerevisiae*	Nucleus
Transcriptional regulator of filamentous growth FLO8	Q59QW5	*Candida albicans*	Nucleus
Transcription factor VHR1	P40522	*Saccharomyces cerevisiae*	Nucleus

bZip transcription factor GAP1	Q6FRZ8	*Candida glabrata*	Nucleus
RNA polymerase I-specific transcription initiation factor RRN5	Q02983	*Saccharomyces cerevisiae*	Nucleus
ATPase synthesis protein 25, mitochondrial	Q6C7D6	*Yarrowia lipolytica*	Mitochondrion inner membrane
Siderophore biosynthesis regulatory protein URBS1	P40349	*Ustilago maydis*	Nucleus
Flap endonuclease 1	A4QS18	*Magnaporthe oryzae*	Nucleus / nucleolus
RNA-silencing factor ers1	O94717	*Schizosaccharomyces pombe*	Cytoplasm
Telomere length regulation protein TEN1	Q07921	*Saccharomyces cerevisiae*	Chromosome / telomeric region
Histone acetyltransferase type B subunit 2	Q6FXI8	*Candida glabrata*	Cytoplasm

Amino acid metabolism

High-affinity glutamine permease	P48813	*Saccharomyces cerevisiae*	Mitochondrion membrane
Vacuolar amino acid transporter 3	P36062	*Saccharomyces cerevisiae*	Vacuole membrane
D-3-phosphoglycerate dehydrogenase 2	P40510	*Saccharomyces cerevisiae*	Cytoplasm

Glutathione hydrolase	W9JMZ4	*Fusarium oxysporum*	Mitochondrion /cytoplasm
PALP domain-containing protein	A0A5C6TN97	*Fusarium oxysporum*	Mitochondrion
Glutathione synthetase	A0A420MCL8	*Fusarium oxysporum*	Nucleus
Pentafunctional AROM polypeptide	A0A2H3TLG9	*Fusarium oxysporum*	Cytoplasm
Kynurenine formamidase	Q04066	*Saccharomyces cerevisiae*	Cytoplasm
Methylthioribose-1-phosphate isomerase	A7TSA5	*Vanderwaltozyma polyspora*	Cytoplasm
Thiamine thiazole synthase	A7EWL8	*Sclerotinia sclerotiorum*	Cytoplasm

<u>Stress and Defense Response</u>

Heat shock protein homolog SSE1	P32589	*Saccharomyces cerevisiae*	Cytoplasm
Membrane-anchored lipid-binding protein YSP2	Q06681	*Saccharomyces cerevisiae*	Endoplasmic reticulum membrane
Heat shock protein SSA4	P22202	*Saccharomyces cerevisiae*	Cytoplasm
6-methylsalicylic acid synthase	P22367	*Penicillium patulum*	Cytoplasm

Mechanosensitive ion channel protein Msy2	O14050	*Schizosaccharomyces pombe*	Endoplasmic reticulum membrane
Protein SIP5	Q759M1	*Ashbya gossypii*	Cytoplasm
Dicer-like protein 2	A1D9Z6	*Neosartorya fischeri*	Cytoplasm
Stress response protein nst1	Q7S8V3	*Neurospora crassa*	Cytoplasm
Cytochrome c peroxidase, mitochondrial	Q6BKY9	*Debaryomyces hansenii*	Mitochondrion
Transcription regulation			
Protein hir1	Q0CQ54	*Aspergillus terreus*	Nucleus
Other Metabolites			
Inactive deaminase YBR284W	P38150	*Saccharomyces cerevisiae*	Cytoplasm/Membrane
Urea amidolyase	P32528	*Saccharomyces cerevisiae*	Cytoplasm
2,5-diamino-6-ribosylamino-4(3H) -pyrimidinone 5'-phosphate reductase	P33312	*Saccharomyces cerevisiae*	Cytoplasm
FAD_binding_3 domain-containing Protein	A0A6M7Z3Q6	*Fusarium oxysporum*	Mitochondrion
Adenosylmethionine decarboxylase	W9HY08	*Fusarium oxysporum*	Cytoplasm
Molybdopterin synthase catalytic subunit	A2R3H4	*Aspergillus niger*	Cytoplasm
Adenylate cyclase	P23466	*Lachancea kluyveri*	Nucleus

Hydroxamate-type ferrichrome siderophore peptide synthetase	Q9P7T1	*Schizosaccharomyces pombe*	Cytoplasm
3-ketosteroid 1-dehydrogenase helE	Q4WR24	*Neosartorya fumigata*	Mitochondrion
Folic acid synthesis protein FOL1	P53848	*Saccharomyces cerevisiae*	Mitochondrion membrane
FAD-dependent monooxygenase sdgC	A0A1U8QHS4	*Emericella nidulans*	Membrane
Unknown			
Protein RFS1	P38234	*Saccharomyces cerevisiae*	Cytoplasm
PX domain-containing protein YPR097W	Q06839	*Saccharomyces cerevisiae*	Cytoplasm
Protein FMP27, mitochondrial	Q06179	*Saccharomyces cerevisiae*	Mitochondrion
BZIP domain-containing protein	A0A6M7Z7J4	*Fusarium oxysporum*	Nucleus
ABC1 domain-containing protein	A0A6M7Z9K2	*Fusarium oxysporum*	Nucleus
AMP-binding domain-containing protein	W9KNE1	*Fusarium oxysporum*	Cytoplasm
Mtf2 domain-containing protein	A0A2H3T9L8	*Fusarium oxysporum*	Mitochondrion
gag_pre-integrs domain-containing protein	A0A420PAX1	*Fusarium oxysporum*	Nucleus
SCP domain-containing protein	A0A6M7ZUM5	*Fusarium oxysporum*	Nucleus
HTH APSES-type domain-containing protein	W9KJX9	*Fusarium oxysporum*	Nucleus
Spc7 domain-containing protein	W9LBX5	*Fusarium oxysporum*	Nucleus

HET domain-containing protein	X0B8E0	*Fusarium oxysporum*	Nucleus/Cytoplasm
Isochorismatase domain-containing protein	W9K8Q9	*Fusarium oxysporum*	Cytoplasm
2EXR domain-containing protein	A0A6M7ZEK6	*Fusarium oxysporum*	Unknown
DJ-1_PfpI domain-containing protein	A0A6M7YN02	*Fusarium oxysporum*	Unknown
Abhydrolase_3 domain-containing protein	A0A2H3GEN0	*Fusarium oxysporum*	Cytoplasm
Cytochrome b5 heme-binding domain-containing protein	A0A420STL5	*Fusarium oxysporum*	Nucleus
AB hydrolase-1 domain-containing protein	W9JP57	*Fusarium oxysporum*	Cytoplasm
Homeobox domain-containing protein	A0A6M7YN32	*Fusarium oxysporum*	Nucleus
SH3 domain-containing protein	X0IL15	*Fusarium oxysporum*	Integral component of membrane
FAD-binding PCMH-type domain-containing protein	A0A6M7YWV0	*Fusarium oxysporum*	Endoplasmic
Deacetylase sirtuin-type domain-containing protein	A0A6M7ZUM4	*Fusarium oxysporum*	Nucleus

NB-ARC domain-containing protein	W9KX17	*Fusarium oxysporum*	Cytoplasm
5'-deoxynucleotidase	A0A6M7ZG25	*Fusarium oxysporum*	Cytoplasm / Mitochondrion
DNA helicase	W9IMG1	*Fusarium oxysporum*	Nucleus
Dimer_Tnp_hAT domain-containing protein	A0A8H6GUX8	*Fusarium oxysporum*	Cytoplasm / nucleus
J domain-containing protein	A0A8H5AIT8	*Fusarium oxysporum*	Membrane
Maintenance of telomere capping protein 2	C5E1C0	*Zygosaccharomyces rouxii*	Unknown
Membrane-bound O-acyltransferase GUP1	Q7Z888	*Millerozyma farinosa*	Cell membrane
Baeyer-Villiger monooxygenase nsrF	A0A2I1C3U2	*Aspergillus novofumigatus*	Cytoplasm
Cytochrome P450 monooxygenase 98	F1SY91	*Postia placenta*	Membrane
Altered inheritance of mitochondria protein 41, mitochondrial	A5DKQ3	*Meyerozyma guilliermondii*	Mitochondrion
Protein PAR32	Q12515	*Saccharomyces cerevisiae*	Cytoplasm
Nonribosomal peptide synthetase 4	I1RF49	*Gibberella zeae*	Endoplasmic reticulum
Sorbose reductase homolog SOU2	P87218	*Candida albicans*	Cytoplasm

pH-response regulator protein palF/RIM8	Q52FM1	*Magnaporthe oryzae*	Unknown
T-complex protein 1 subunit eta	Q8SR53	*Encephalitozoon cuniculi*	Cytoplasm
Monooxygenase AacuP	A0A1L9WLD7	*Aspergillus aculeatus*	Cytoplasm
FK506-binding protein 4	G0SC91	*Chaetomium thermophilum*	Nucleus
Topoisomerase I damage affected protein 2	A7TTT7	*Vanderwaltozyma polyspora*	Cytoplasm
Cytochrome P450 monooxygenase orf6	A0A068ACU3	*Eupenicillium brefeldianum*	Membrane
